# CAR T cell manufacturing from naive/stem memory T lymphocytes enhances antitumor responses while curtailing cytokine release syndrome

**DOI:** 10.1172/JCI150807

**Published:** 2022-06-15

**Authors:** Silvia Arcangeli, Camilla Bove, Claudia Mezzanotte, Barbara Camisa, Laura Falcone, Francesco Manfredi, Eugenia Bezzecchi, Rita El Khoury, Rossana Norata, Francesca Sanvito, Maurilio Ponzoni, Beatrice Greco, Marta Angiola Moresco, Matteo G. Carrabba, Fabio Ciceri, Chiara Bonini, Attilio Bondanza, Monica Casucci

**Affiliations:** 1 Innovative Immunotherapies Unit, Division of Immunology, Transplantation and Infectious Diseases,; 2 Experimental Hematology Unit - Division of Immunology, Transplantation and Infectious Diseases,; 3 Center for Omics Sciences,; 4 San Raffaele Telethon Institute for Gene Therapy (SR-TIGET), and; 5 Pathology Unit, Division of Experimental Oncology, IRCCS San Raffaele Scientific Institute, Milan, Italy.; 6 Vita-Salute San Raffaele University, Milan, Italy.; 7 Department of Hematology and Stem Cell Transplantation - IRCCS San Raffaele Scientific Institute, Milan, Italy.

**Keywords:** Immunology, Therapeutics, Cancer immunotherapy, Immunotherapy, T cells

## Abstract

Chimeric antigen receptor (CAR) T cell expansion and persistence represent key factors to achieve complete responses and prevent relapses. These features are typical of early memory T cells, which can be highly enriched through optimized manufacturing protocols. Here, we investigated the efficacy and safety profiles of CAR T cell products generated from preselected naive/stem memory T cells (T_N/SCM_), as compared with unselected T cells (T_BULK_). Notwithstanding their reduced effector signature in vitro, limiting CAR T_N/SCM_ doses showed superior antitumor activity and the unique ability to counteract leukemia rechallenge in hematopoietic stem/precursor cell–humanized mice, featuring increased expansion rates and persistence together with an ameliorated exhaustion and memory phenotype. Most relevantly, CAR T_N/SCM_ proved to be intrinsically less prone to inducing severe cytokine release syndrome, independently of the costimulatory endodomain employed. This safer profile was associated with milder T cell activation, which translated into reduced monocyte activation and cytokine release. These data suggest that CAR T_N/SCM_ are endowed with a wider therapeutic index compared with CAR T_BULK_.

## Graphical abstract

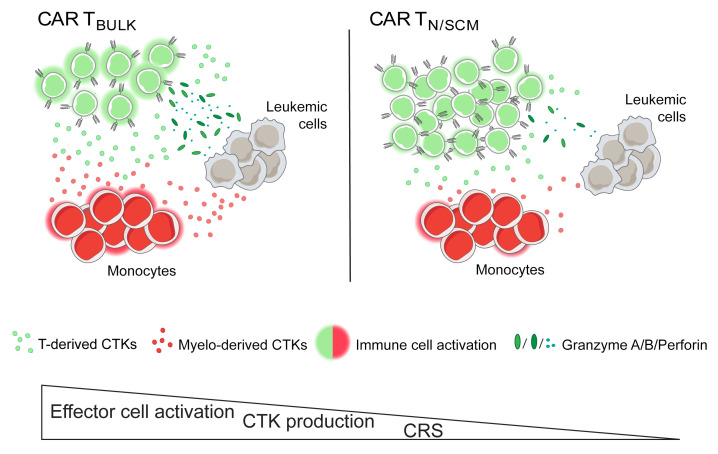

## Introduction

CAR T cell therapy has considerably changed the landscape of treatment options for B cell malignancies, leading to the recent approval of the first CAR T cell products for treating cancer (1–8). However, frequent relapses in treated patients, together with inability to achieve complete remission in certain disease types (4, 9–11), highlight the need of further potentiating this therapeutic strategy (12). In addition, manifestation of severe toxicities, such as cytokine release syndrome (CRS) and immune effector cell–associated neurotoxicity syndrome (ICANS), still needs to be efficiently counteracted without limiting functionality (13, 14).

Extensive clinical experience has indicated that primary objective responses are strictly associated with the level of CAR T cell expansion early after infusion, while long-term persistence is required to prevent relapses (3, 15, 16). At the same time, however, factors associated with enhanced CAR T cell proliferation in vivo, such as higher CAR T cell expansion peaks, as well as larger tumor burdens, cyclophosphamide-fludarabine lymphodepletion regimens, and greater CAR T cell doses strongly correlate with the incidence and severity of CRS and ICANS (13, 17–19).

Among others, intrinsic T cell properties and composition of the infused T cell product have been reported to significantly shape CAR T cell fitness (20, 21). Indeed, T cells exist in a wide range of interconnected differentiation statuses, extensively differing in terms of proliferative capacity, self-renewal capabilities, and long-term survival (15, 20, 22). In this regard, accumulating evidence in mice and humans suggests that T cell differentiation negatively correlates with long-term antitumor activity, with early memory T cells holding the most favorable features (15, 22). Accordingly, T cells from patients with large B cell lymphoma and chronic lymphocytic leukemia (CLL) who responded to CD19 CAR T cells were found enriched in gene expression profiles involved in early memory, or were rather the result of a single central memory T cell (T_CM_) clone derived from a TET2-targeted insertional mutagenesis event, as observed in a patient with CLL (4, 6, 23, 24).

Recently, the identification of stem memory T cells (T_SCM_), embodying the apex of the T cell differentiation hierarchy (15, 25, 26), paved the way for the employment of this T cell source for cancer immunotherapy. Since T_SCM_ are extremely rare in the peripheral circulation, several efforts have been dedicated to the development of robust manufacturing protocols capable of generating and expanding this cell subset in vitro (15, 20, 26–30). In particular, it has been reported that preselection of naive T cells (T_N_) before manipulation represents a crucial step for enriching T_SCM_ (20, 26, 30). Indeed, the presence of more differentiated T cells during T_N_ stimulation has been reported to accelerate their functional, transcriptional, and metabolic differentiation, owing to intercellular quorum-sensing mechanisms (31). Accordingly, preselection of T_N_ improves the features of the final T cell product, which has proven to be intrinsically less activated at the end of culture but superior in its ability to expand and differentiate into effectors able to mediate a potent xenogeneic graft-versus-host disease (26). Although the superior antitumor activity of T_N_-derived CAR T cells has already been profiled, a thorough evaluation of their functional behavior in complex animal models is still lacking, especially regarding toxicity, which is particularly warranted due to the typical superior expansion capability of T_N/SCM_.

In this work, we revised the hematopoietic stem/precursor cell–humanized (HSPC-humanized) mouse model we recently developed (32), which is capable of recapitulating CAR T cell–related toxicities at the pathophysiological level, to investigate the efficacy and safety profiles of CAR T cells generated from preselected T_N_/_SCM_ precursors. In line with previous findings, CAR T_N/SCM_ displayed superior capability in protecting mice from leukemia rechallenge, compared with CAR T cells generated from unselected T cells. Surprisingly, however, such increased potency and higher expansion were associated with limited incidence of severe CRS (sCRS) and neurotoxicity, uncovering possible mechanisms accounting for these toxicities. Among these, we found that CAR T cells actively shape monocyte activation and that CAR T_N/SCM_ are more proficient at fine-tuning the dynamic equilibrium that regulates monocyte-derived cytokine release, rendering these cells a valuable option to widen the therapeutic index of current CAR T cell therapies.

## Results

### CAR T_N/SCM_ display a less pronounced effector signature compared with CAR T_BULK_ in vitro.

**Figure 1:**
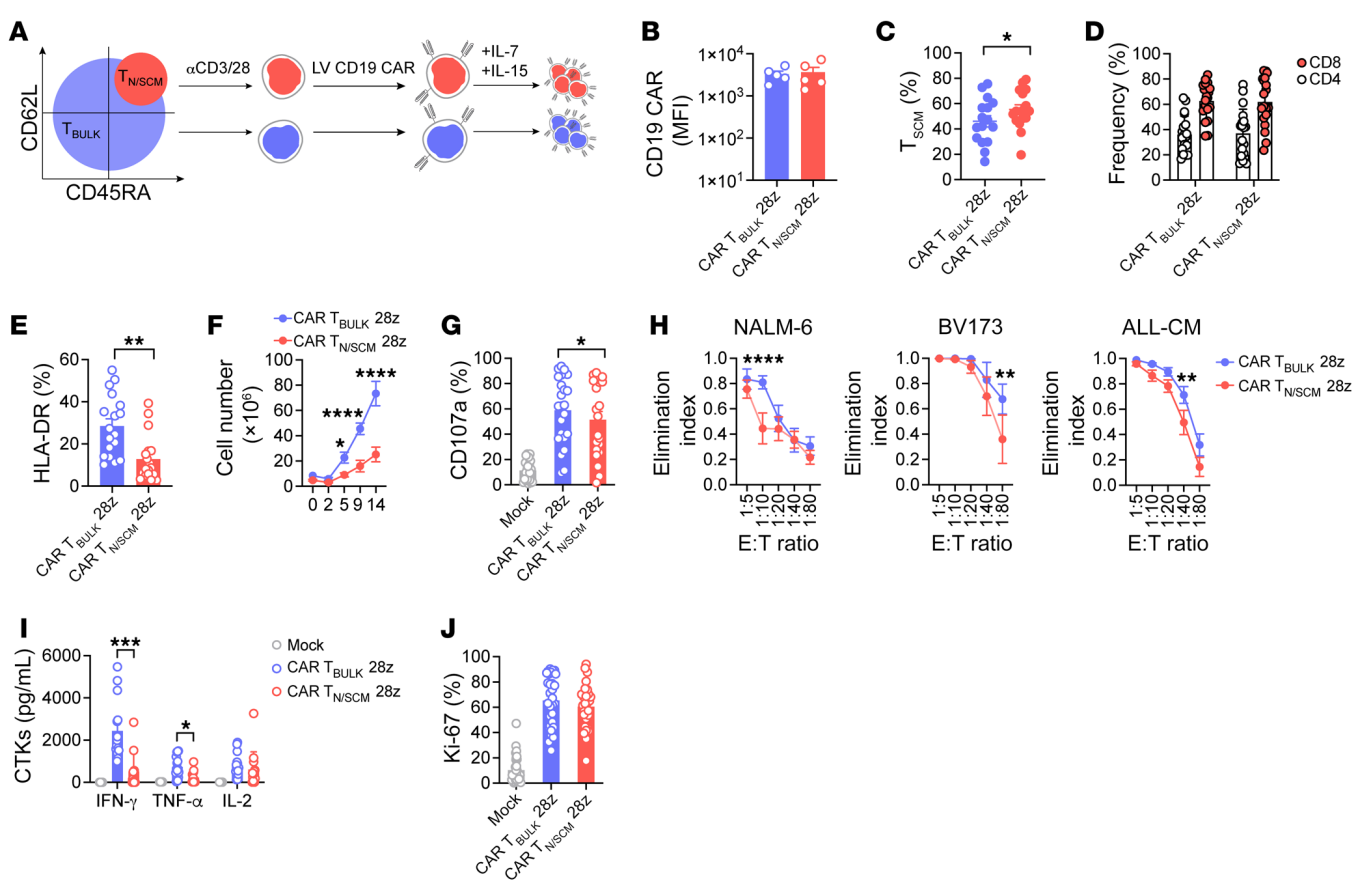
CAR T_N/SCM_ display an indolent effector signature in vitro. (**A**) Schematic representation of CAR T cell manufacturing. Briefly, double-positive CD62L^+^CD45RA^+^ T_N/SCM_ cells were isolated by FACS and bulk unselected T cells (T_BULK_) were employed as control. T_N/SCM_ and T_BULK_ were activated with TransAct (anti-CD3/anti-CD28 [αCD3/28]), transduced with a lentiviral vector (LV) encoding a CD19.28z CAR, and expanded in culture with IL-7 and IL-15. (**B**) CD19.28z CAR expression (mean fluorescence intensity, MFI; *n =* 5), (**C**) T_SCM_ enrichment (*n =* 16), (**D**) CD4^+^/CD8^+^ ratio (*n =* 20), and (**E**) HLA-DR expression (percentage of positive cells, *n =* 18) at the end of CAR T cell manufacturing. (**F**) Fold expansion at different days of culture (*n =* 12). (**G**) Degranulation assay performed by coculturing CAR T_N/SCM_, CAR T_BULK_, and Mock control with CD19^+^ targets for 24 hours (*n =* 14 donors challenged against NALM-6, BV173, and ALL-CM CD19^+^ target cell lines). (**H**) Killing activity expressed as elimination index (see Methods) and measured by coculturing CAR T cells with CD19^+^ tumor cells for 4 days at different effector/target (E:T) ratios (*n =* 15 for CAR T_BULK_, *n =* 14 for CAR T_N/SCM_ against NALM-6 cell line; *n =* 7 for CAR T_BULK_, *n =* 6 for CAR T_N/SCM_ against BV173 cell line; *n =* 8 for CAR T_N/SCM_, *n =* 9 for CAR T_BULK_ against ALL-CM cell line). (**I**) Cytokine (CTK) production after 24-hour coculture of T cells with CD19^+^ tumor cells at a 1:10 E:T ratio (*n =* 5 donors challenged against NALM-6, BV173, and ALL-CM cell lines). (**J**) T cell proliferation after 4-day coculture with CD19^+^ tumor cells, measured by intracellular staining of Ki-67 (*n =* 15 donors challenged against NALM-6, BV173, and ALL-CM cell lines). Data are represented as mean ± SEM or mean ± SEM together with overlapping scattered values. *P < 0.05, **P < 0.01, ***P < 0.001, ****P < 0.0001 by paired *t* test (**B**–**E**, **G**, and **L**) or 2-way ANOVA (**F**, **H**, and **I**).

With the aim of determining whether preselection of early memory subsets as starting sources for manufacturing could enhance the therapeutic potential of CAR T cells, we isolated CD62L^+^CD45RA^+^ T_N/SCM_ by FACS with a purity of approximately 99.1% and employed bulk unselected T cells (T_BULK_) for comparison. Both T_N/SCM_ and T_BULK_ were activated with the TransAct nanomatrix, transduced to express a CD28-costimulated CD19 CAR and expanded with IL-7 and IL-15 (Figure 1A), according to a protocol capable per se of preserving T cell fitness.

Phenotypic characterization at the end of culture pointed out superimposable expression levels of the CAR molecule (Figure 1B) and the truncated low-affinity nerve growth factor receptor (ΔLNGFR) marker gene (Supplemental Figure 1A; supplemental material available online with this article; https://doi.org/10.1172/JCI150807DS1) in the 2 cell products. Conversely, a higher proportion of T_SCM_ was observed in CAR T_N/SCM_ compared with CAR T_BULK_ (Figure 1C), together with a reduction in effector memory T cells (T_EM_) (Supplemental Figure 1B), even though a similar CD4^+^/CD8^+^ cell ratio was maintained (Figure 1D). In addition, a lower activation profile in terms of HLA-DR expression and a reduced expansion during manufacturing were characteristic of CAR T_N/SCM_ compared with CAR T_BULK_ (Figure 1, E and F).

To evaluate whether the 2 CAR T cell products exhibited different functional capabilities, we challenged them against CD19^+^ leukemia cell lines. CAR T_N/SCM_ cells displayed a slightly reduced degranulation capability (Figure 1G) and cytotoxic potential (Figure 1H), and were associated with lower production of proinflammatory cytokines with respect to CAR T_BULK_ (Figure 1I). In contrast, we observed a similar proliferation response between the 2 CAR T cell populations after short-term in vitro coculture with tumor cells (Figure 1J). Interestingly, even though coexpression of PD-1, LAG-3, and TIM-3 inhibitory receptors (IRs) was similar after stimulation with CD19^+^ targets, the overall exhausted-like status of CAR T_N/SCM_ was reduced compared with CAR T_BULK_, as displayed by lower cumulative expression levels of IRs (Supplemental Figure 1C).

These data indicate that the 2 CAR T cell products are phenotypically and functionally different, with CAR T_BULK_ showing a more pronounced effector signature than CAR T_N/SCM_.

### CAR T_N/SCM_ are uniquely able to elicit recall antitumor responses in HSPC-humanized mice.

Compared with the standard NSG mouse model, the HSPC-humanized mouse model in triple-transgenic SGM3 mice is known to better support human health and tumor hematopoiesis (32, 33). In this model, we previously reported that the presence of human myeloid cells is crucial to trigger CRS and neurotoxicity (32). We here hypothesized that this complex human network, which includes human immune cells and cytokines that are absent in classical xenograft mouse models, could be instrumental to better appreciate the fitness of different CAR T cell products in terms of both antitumor potential and safety profiles.

We therefore reconstituted SGM3 mice with human cord blood CD34^+^ cells and infused humanized mice (HuSGM3) with NALM-6 leukemia cells. Leukemia-bearing mice were then treated with CAR T_N/SCM_ or CAR T_BULK_ and monitored for T cell expansion, tumor progression, and overt toxicities. In this context, leukemia control was equally achieved by both CAR T_N/SCM_ and CAR T_BULK_, even though CAR T cell expansion was higher when looking at CAR T_N/SCM_–treated mice (Supplemental Figure 2, A and B). Notably, in this experimental setting characterized by a low tumor burden, mice did not experience sCRS, as indicated by only moderate and reversible weight loss and modest elevation of serum levels of IL-6 and serum amyloid A (SAA), a murine homolog of the human CRS biomarker C-reactive protein (ref. 32 and Supplemental Figure 2, C and D).

**Figure 2:**
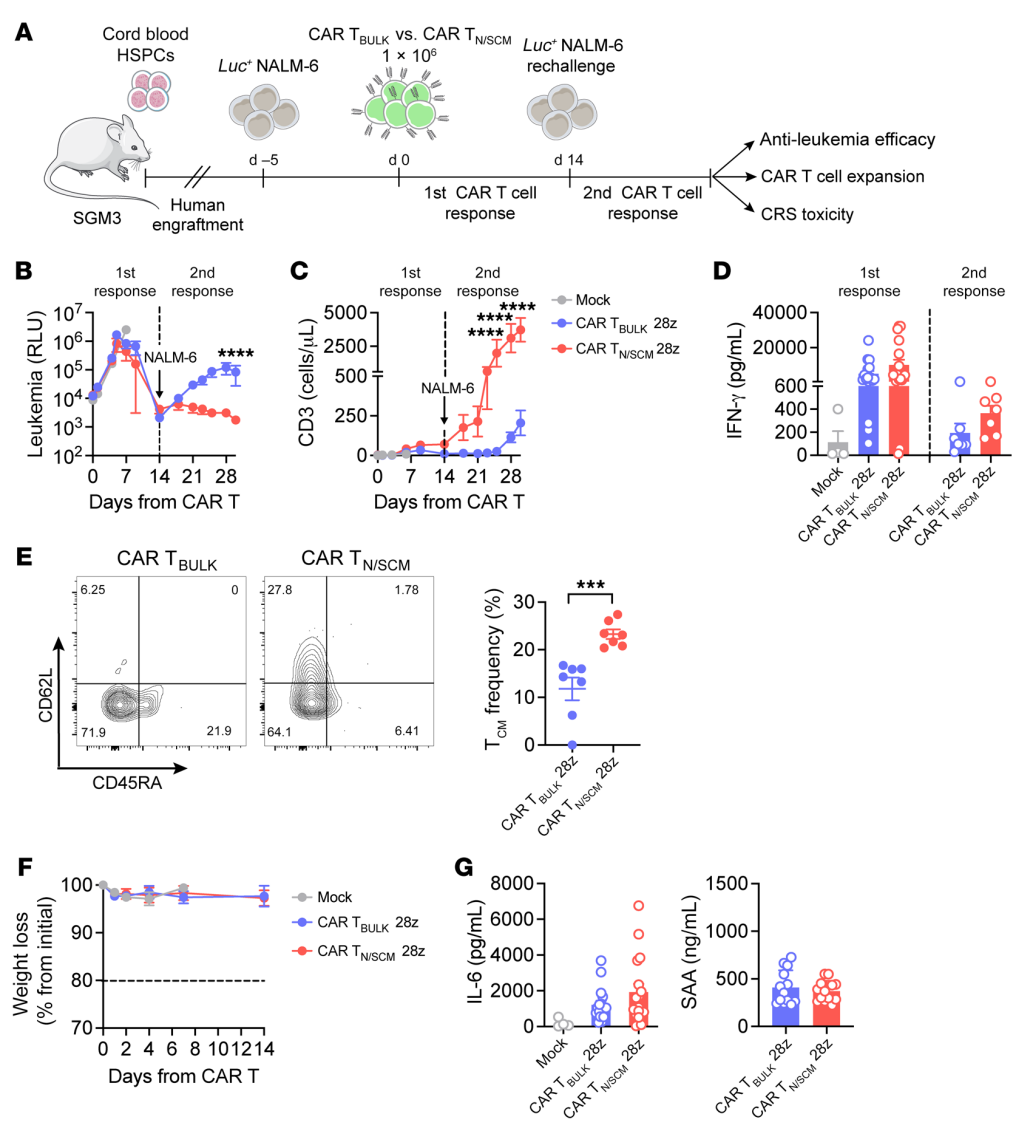
CAR T_N/SCM_ display superior antitumor activity and expansion in HuSGM3 mice. (**A**) Schematic representation of the HSPC-humanized mouse model for efficacy testing. SGM3 mice were infused with HSPCs and, after hematopoietic reconstitution, injected with Lucia^+^NGFR^+^ NALM-6 leukemia cells and treated with low doses of CD28-costimulated CAR T_N/SCM_ (*n =* 17), CAR T_BULK_ (*n =* 17), or Mock control (*n =* 7). (**B**) NALM-6–derived bioluminescence signal measured at different time points after treatment and expressed as relative light units (RLU). (**C**) T cell expansion in the peripheral blood of NALM-6–bearing mice measured at different time points after treatment. (**D**) IFN-γ serum levels measured on day 4 after treatment and day 5 after NALM-6 rechallenge. (**E**) T cell memory phenotype of CAR T_BULK_ and CAR T_N/SCM_ on day 14 after treatment. Left panel: Dot plot of 2 representative mice (T_SCM_: CD45RA^+^CD62L^+^; T_CM_: CD45RA^–^CD62L^+^; T_EM_: CD45RA^–^CD62L^–^; T_EMRA_: CD45RA^+^CD62L^–^). Right panel: Frequency of T_CM_ cells in mice from the 2 cohorts (analysis performed for *n =* 7 mice/group). (**F** and **G**) Evaluation of signs and symptoms typical of CRS development in HuSGM3 leukemia–bearing mice after treatment, represented by weight loss (**F**), serum levels of IL-6 (**G**, left), and murine serum amyloid A (SAA; **G**, right). Data are represented as mean ± SEM together with overlapping scattered values. ***P < 0.001; ****P < 0.0001 by 2-way ANOVA (**B**–**D** and **F**) or unpaired *t* test (**E** and **G**).

To further challenge the therapeutic potential of the 2 CAR T cell populations, we performed a similar experiment in HuSGM3 mice, where we injected a lower T cell dose and provided a second tumor rechallenge (Figure 2A). In this setting, CAR T_N/SCM_ showed comparable activity to that of CAR T_BULK_ during the first antitumor response but were uniquely able to elicit recall responses upon leukemia rechallenge (Figure 2B). This improved therapeutic potential was associated with increased CAR T cell expansion rates (Figure 2C), which were evident both in the CD4^+^ and CD8^+^ compartments (Supplemental Figure 2E), and with a trend toward higher release of IFN-γ, especially during the second antitumor response (Figure 2D). In line with our previous observations (34, 35), CD8^+^ CAR T cells were enriched immediately after leukemia encounter in both conditions, while CD4^+^ CAR T cells became prominent at later time points (Supplemental Figure 2F). Notably, 14 days after infusion, CAR T_N/SCM_ contained an increased T_CM_ percentage compared with CAR T_BULK_ (Figure 2E), possibly accounting for their superior and long-lasting therapeutic activity. Even in this setting, no signs of sCRS were detected independently of the CAR T cell population employed, as indicated by absence of weight loss and only moderate elevation of serum IL-6 and SAA (Figure 2, F and G).

Collectively, these results suggest that CAR T_N/SCM_ may induce more durable antitumor responses than CAR T_BULK_, thanks to higher expansion rates and early memory preservation after leukemia encounter.

### Barnes-Hut stochastic neighbor embedding algorithm identifies a best-performing phenotype typical of CAR T_N/SCM_.

The selective enrichment of T_CM_ in mice treated with CAR T_N/SCM_ prompted us to investigate whether the functional differences between the 2 CAR T cell populations could be ascribed to a different phenotype after leukemia encounter in vivo. To answer this question, we performed the same experiment as described in Figure 2A, but with the aim of deepening the phenotypic characterization of CAR T cells after the first response, i.e., at day 14 after CAR T cell infusion. To this aim, we sought to employ an unsupervised approach based on the Barnes-Hut stochastic neighbor embedding (BH-SNE) dimensionality reduction algorithm for data analysis (36–38).

**Figure 3:**
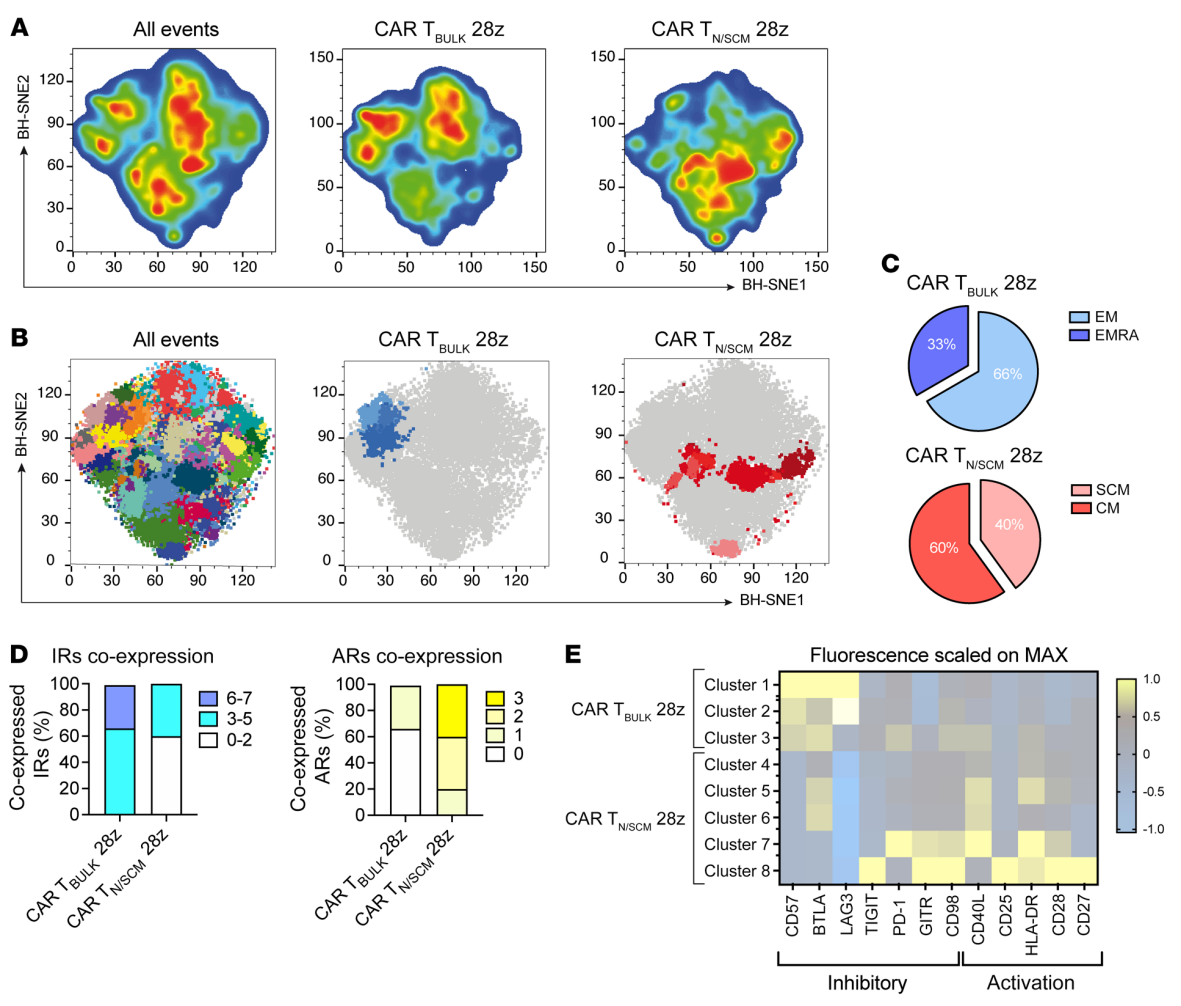
CAR T_N/SCM_ retain an enhanced in vivo fitness after leukemia encounter. SGM3 mice were infused with HSPCs and, after hematopoietic reconstitution, injected with Lucia^+^NGFR^+^ NALM-6 leukemia cells and treated with low doses of CD28-costimulated CAR T_N/SCM_ (*n =* 3) or CAR T_BULK_ (*n =* 5) as described in Figure 2. (**A**) A median of approximately 74,000 CD3^+^ lymphocytes derived from the peripheral blood of both CAR T_N/SCM_– and CAR T_BULK_–treated mice on day 14 after treatment were interrogated by BH-SNE and K-means algorithms. Data were plotted according to BH-SNE1 and BH-SNE2 specifically calculated variables and the events were split into 2 density plots according to the CAR T cell population they belong to. (**B**) CAR T_N/SCM_ and CAR T_BULK_ specifically identified clusters after application of Flow-SOM algorithm to both BH-SNE1 and BH-SNE2 variables. CAR T_N/SCM_– and CAR T_BULK_–specific clusters described in terms of (**C**) T cell memory subset composition, together with (**D**) expression of inhibitory and activation receptors (IRs and ARs). (**E**) Heatmap visualization of both inhibitory and activation receptors expressed by CAR T_N/SCM_– and CAR T_BULK_–specific metaclusters, in which mean fluorescence intensity (MFI) levels were normalized on the basis of the maximum expressed value of each analyzed parameter in the whole examined sample.

As formerly observed, no difference in the capability of controlling leukemia growth was observed between CAR T_N/SCM_ and CAR T_BULK_ (Supplemental Figure 3A). However, unsupervised and stochastic data downscaling, in which approximately 74,000 CD3^+^ lymphocytes were chosen for each file, together with the multidimensionality reduction performed by BH-SNE analysis, revealed the enrichment of clusters in totally distinct areas between CAR T_N/SCM_ and CAR T_BULK_ (Figure 3, A and B). Examination of these clusters, in which a similar distribution of each sample was found (Supplemental Figure 3B), highlighted intrinsic differences in the phenotypic composition of CAR T_N/SCM_ when compared with CAR T_BULK_. Of note, CAR T_N/SCM_ clusters were extremely enriched in T_SCM_ and T_CM_, whereas those concerning CAR T_BULK_ preferentially exhibited a T_EM_ and effector memory CD45RA^+^ (EMRA) phenotype (Figure 3C). Moreover, CAR T_N/SCM_ displayed an activated phenotype, characterized by coexpression of activation markers and limited enrichment of IRs, while CAR T_BULK_ were typified by an exhausted phenotype, coexpressing multiple IRs in the absence of activation markers (Figure 3D). Indeed, the opposed spatial orientation of CAR T_N/SCM_ and CAR T_BULK_ was directed toward the enrichment of either activation receptors or IRs, respectively, as evidenced by heatmap visualization (Figure 3E).

In conclusion, this unsupervised approach revealed that CAR T_N/SCM_ are endowed with enhanced in vivo fitness, which relies on an improved preservation of early memory cells, higher activation, and lower exhaustion.

### CAR T_N/SCM_ are intrinsically less prone to causing sCRS.

**Figure 4:**
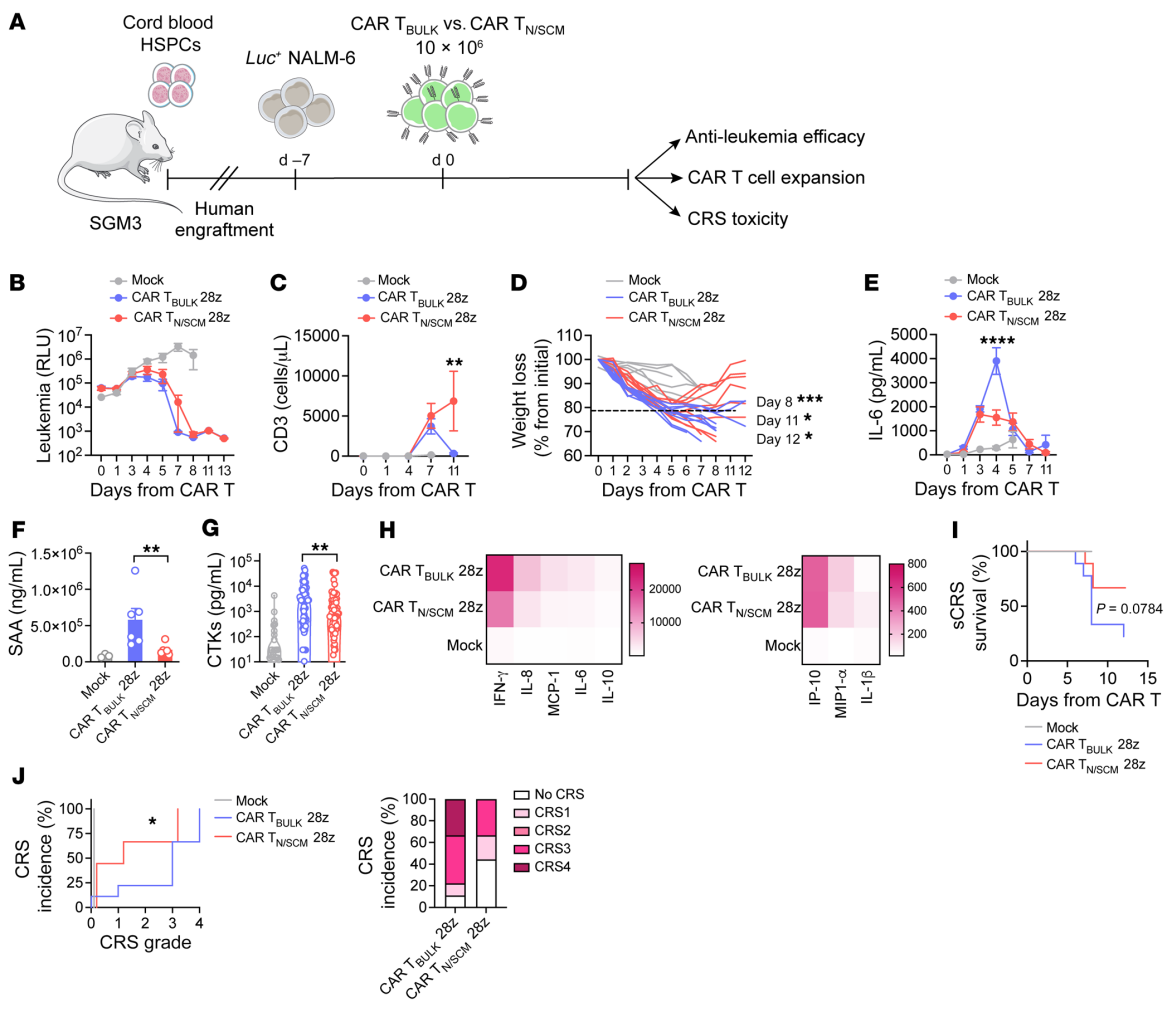
CAR T_N/SCM_ are less prone to induce severe CRS. (**A**) SGM3 mice were infused with HSPCs and, after hematopoietic reconstitution (HuSGM3), injected with Lucia^+^NGFR^+^ NALM-6 leukemia cells. When a high tumor burden was reached, mice were treated with high doses of CD28-costimulated CAR T_N/SCM_ (*n =* 9), CAR T_BULK_ (*n =* 9), or Mock control (*n =* 6). (**B**) NALM-6–derived bioluminescence signal measured at different time points after treatment and expressed as relative light units (RLU). (**C**) T cell expansion in the peripheral blood of mice, (**D**) weight loss evaluation, and (**E**) IL-6 serum levels at different time points after treatment. (**F**) Serum amyloid A (SAA) levels 24 hours after T cell infusion (*n =* 6 for CAR T_BULK_, *n =* 6 for CAR T_N/SCM_, *n =* 3 for Mock). (**G**) Peak serum cytokine (CTK) levels and (**H**) heatmap visualization of peak serum cytokine levels on day 4 after treatment. Data are represented as the mean ± SEM and values are scaled according to a graded-color range depending on relative minimum and maximum levels. (**I**) Severe CRS–related (sCRS-related) Kaplan-Meier survival analysis of mice. (**J**) CRS grading. Left panel: Kaplan-Meier curves. Right panel: Histograms summarizing CRS grading. Data are represented as mean ± SEM together with overlapping scattered values and box and violin plots. *P < 0.05, **P < 0.01, ***P < 0.001, ****P < 0.0001 by 2-way ANOVA (**B**–**E**), unpaired *t* test (**F** and **G**), Mantel-Cox 2-sided log-rank test (**I**), or Gehan-Breslow-Wilcoxon test (**J**).

Concerned about the higher expansion rate displayed by CAR T_N/SCM_, which may theoretically increase their toxic potential, we modified the previous experimental setting in HuSGM3 mice to exacerbate their intrinsic capability to elicit sCRS. Since such an adverse event is known to be associated with both tumor burden and the level of CAR T cell expansion upon infusion (14, 39, 40), we increased leukemia load and CAR T cell dose by approximately 1 log (Figure 4A). In these conditions, CAR T_N/SCM_ and CAR T_BULK_ were mutually able to control leukemia growth, even though CAR T_N/SCM_ showed slightly slower kinetics of tumor clearance (Figure 4B). Despite similar antitumor activity, CAR T_N/SCM_ proliferated more than CAR T_BULK_, confirming that these cells are endowed with a superior expansion potential in vivo (Figure 4C). In this context, increased proliferation of CAR T_N/SCM_ was evident mainly in the CD4^+^ compartment, while the CD4^+^/CD8^+^ ratio tended toward CD8^+^ at early time points and toward CD4^+^ later on (Supplemental Figure 4, A and B). Strikingly, however, while the majority of mice treated with CAR T_BULK_ experienced severe, irreversible weight loss, most animals treated with CAR T_N/SCM_ eventually recovered from toxicity (Figure 4D). Similarly to what was observed in patients and in previous preclinical studies (14, 19, 32, 41), CRS development in mice was associated with elevation of serum IL-6 and SAA, which were both higher in mice treated with CAR T_BULK_ compared with mice that received CAR T_N/SCM_ (Figure 4, E and F). Besides IL-6, a wide array of other proinflammatory cytokines released by immune components in concert with activated CAR T cells was analyzed and, once again, overall cytokine levels were lower in mice receiving CAR T_N/SCM_ than in those infused with CAR T_BULK_ (Figure 4G). Heatmap visualization of cytokine levels and composition confirmed this picture and revealed greater amounts of myeloid-derived cytokines, including IP-10, IL-8, and MCP-1 in CAR T_BULK_–treated mice compared with CAR T_N/SCM_ (Figure 4H). Accordingly, a higher proportion of mice that received CAR T_BULK_ succumbed to sCRS as compared with mice treated with CAR T_N/SCM_ (Figure 4I). In order to more precisely stratify CRS development, we then considered multiple parameters, i.e., weight loss, death event, and myeloid-derived cytokine levels to generate an algorithm that assigns to each mouse a CRS score and allows recapitulation of the grading system employed in patients. By applying this algorithm, we observed that none of the mice treated with CAR T_N/SCM_ developed grade 4 CRS, which conversely was observed in 33% of mice treated with CAR T_BULK_ (Figure 4J). Moreover, while absence of CRS was observed only in the 11% of CAR T_BULK_–treated mice, this proportion increased to 44% in the cohort infused with CAR T_N/SCM_, suggesting that this cell product has a lower potential to cause CRS.

With the aim of evaluating signs of neurotoxicity concomitant with CRS development, mouse brains were collected at sacrifice and subjected to histopathological evaluation. Impressively, 3 out of the 5 CAR T_BULK_–treated mice presented multifocal brain hemorrhages (42), whereas in the group infused with CAR T_N/SCM_ only 1 mouse showed a small hemorrhagic focus (Supplemental Figure 4C).

Taken together, these results indicate that, despite a greater expansion potential, CAR T_N/SCM_ are less prone to trigger detrimental CRS than CAR T_BULK_, displaying a better balance between efficacy and safety profiles. Since before treatment the absolute counts of circulating monocytes, which are crucial for CRS pathogenesis (32, 41), were superimposable in the 2 groups (Supplemental Figure 4D), the reasons for differential toxicity must be sought in the intrinsic biology of the 2 CAR T cell populations.

### CAR T_N/SCM_ are intrinsically less able to trigger sCRS independently of CAR costimulation, by lowering monocyte activation and cytokine production.

The data shown until now refer to CAR T cells incorporating a CD28 costimulatory domain. Aiming to assess whether the reduced toxic profile is an intrinsic property of CAR T cell products generated from T_N/SCM_, we transduced either T_N/SCM_ or T_BULK_ with a 4-1BB–costimulated CAR. Even in this case, CAR expression levels were similar and the proportion of early memory subsets was higher in CAR T_N/SCM_ compared with CAR T_BULK_ (Supplemental Figure 5, A and B), while the CD4^+^/CD8^+^ ratio was similar (Supplemental Figure 5C). In addition, CAR T_N/SCM_ were characterized by a lower activation profile (Supplemental Figure 5D) and reduced expansion in culture (Supplemental Figure 5E).

**Figure 5:**
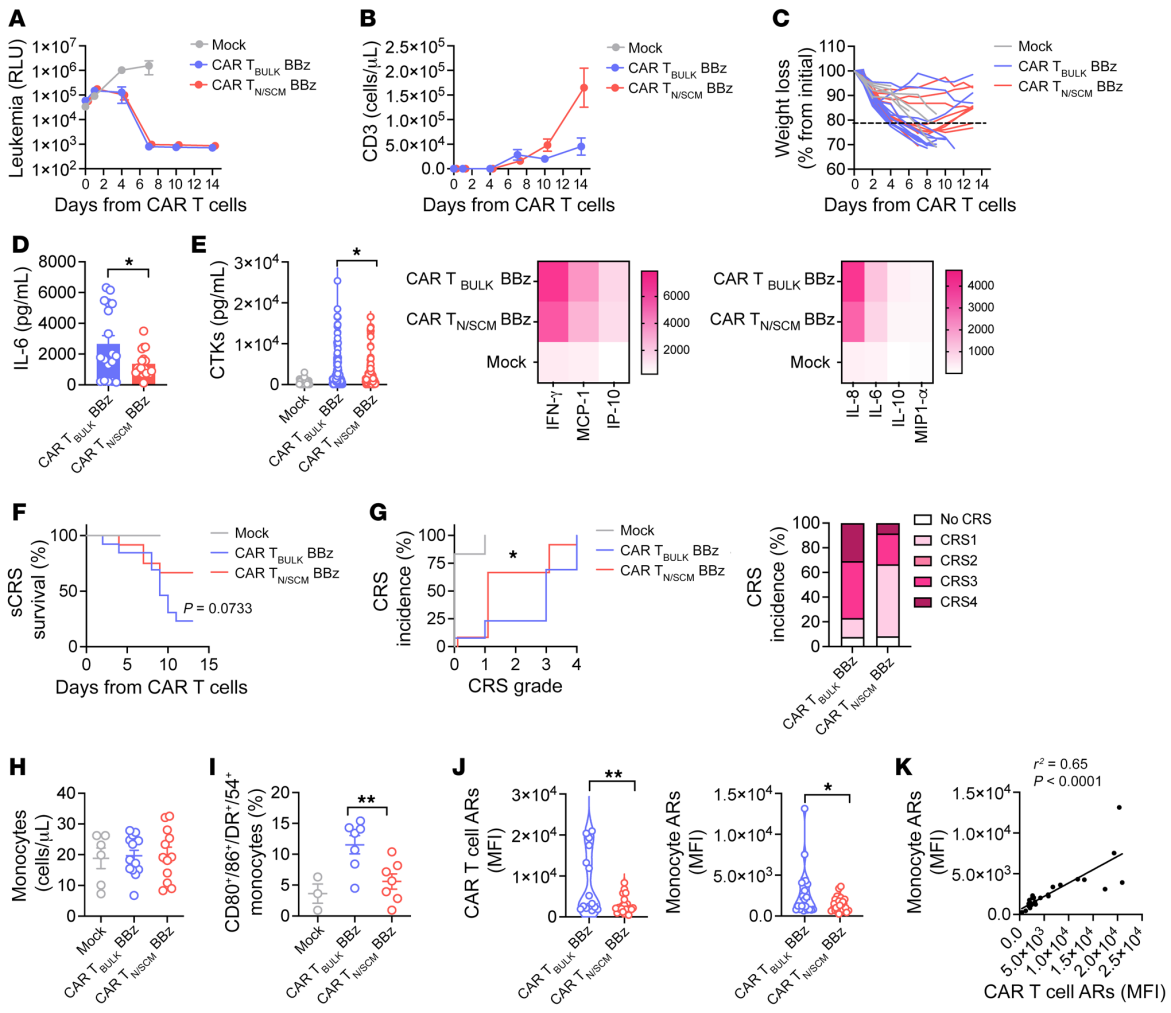
CAR T_N/SCM_ are less toxic, independently of the costimulation provided. Experiments were conducted as described in Figure 4A but with CAR T cells carrying the 4-1BB costimulatory domain. (**A**) NALM-6–derived bioluminescence signal measured at different time points after treatment and expressed as relative light units (RLU) (*n =* 13 for CAR T_BULK_, *n =* 12 for CAR T_N/SCM_, *n =* 6 for Mock). (**B**) T cell expansion in the peripheral blood of mice. (**C**) Weight loss evaluation at different time points after treatment. (**D** and **E**) IL-6 and other cytokine (CTK) serum levels, with their heatmap visualization, on day 4 after treatment (*n =* 18 for CAR T_BULK_, *n =* 19 for CAR T_N/SCM_, *n =* 6 for Mock). (**F**) Severe CRS–related (sCRS-related) Kaplan-Meier survival analysis of mice. (**G**) CRS grading. Left panel: Kaplan-Meier curves. Right panel: Histograms summarizing CRS grading. (**H**) Monocyte absolute number immediately before T cell infusion (*n =* 13 for CAR T_BULK_, *n =* 12 for CAR T_N/SCM_, *n =* 6 for Mock). (**I**) Percentage of activated monocytes coexpressing CD80, CD86, CD54, and HLA-DR activation receptor markers (ARs) 1 day after treatment (*n =* 7 for CAR T_BULK_ and CAR T_N/SCM_, *n =* 3 for Mock). (**J**) Evaluation of AR upregulation on CAR T cells (CD54, CD86) and monocytes (CD54, CD86, CD163) expressed as MFI on day 1 after treatment (*n =* 11 for CD54 and *n =* 7 for CD86 evaluated on CAR T_N/SCM_, *n =* 9 for CD54 and *n =* 6 for CD86 evaluated on CAR T_BULK_, *n =* 6 for CD163 in the CAR T_BULK_ cohort, *n =* 7 for CD163 in the CAR T_N/SCM_ cohort). (**K**) Correlation between CAR T cell and monocyte activation statuses on day 1 after treatment. Data are represented as box and violin plots, mean ± SEM together with overlapping scattered values, or scaled according to a graded-color range depending on relative minimum and maximum levels, when referring to the heatmap. *P < 0.05, **P < 0.01 by 2-way ANOVA (**A**–**C**), unpaired *t* test (**D**, **E**, and **H**–**J**), Mantel-Cox 2-sided log-rank test (**F**), or Gehan-Breslow-Wilcoxon test (**G**).

We next evaluated the safety profile of 4-1BB–costimulated CAR T cells in the same model employed in Figure 4A, including high leukemia burdens and CAR T cell doses. Both CAR T cell populations were equally able to control leukemia growth (Figure 5A), but CAR T_N/SCM_ featured increased CAR T cell expansion rates compared with CAR T_BULK_ (Figure 5B and Supplemental Figure 5, F and G). Like their CD28z counterpart, CAR T_N/SCM_–treated mice also experienced less severe weight loss compared with mice that received CAR T_BULK_ (Figure 5C), together with reduced serum levels of IL-6 (Figure 5D) and other inflammatory cytokines (Figure 5E). Along with this, sCRS-related survival rates in mice infused with CAR T_N/SCM_ were significantly improved compared with CAR T_BULK_ (Figure 5F). Accordingly, the incidence of grade 3 and 4 CRS was significantly higher in the CAR T_BULK_ population than in the CAR T_N/SCM_ cohort (Figure 5G), where grade 1 CRS was rather prevalent.

Intrigued by the enhanced safety profile of CAR T_N/SCM_ despite higher expansion rates, we analyzed the activation profile of monocytes and CAR T cells in these mice. Strikingly, being provided with similar monocyte counts before treatment (Figure 5H), the day after T cell infusion we observed a lower fraction of monocytes coexpressing activation markers, such as CD80, CD86, HLA-DR, and CD54 in mice treated with CAR T_N/SCM_ compared with mice that received CAR T_BULK_ (Figure 5I). Accordingly, the cumulative expression levels of activation markers in CAR T cells and monocytes were reduced in the CAR T_N/SCM_ cohort compared with CAR T_BULK_ (Figure 5J). Finally, a positive correlation between CAR T cell and monocyte activation levels was observed in vivo (Figure 5K).

Overall, these in vivo data show that CAR T_N/SCM_, while displaying a higher expansion capability, are characterized by a lower potential to cause detrimental toxicities, thanks to lower activation levels immediately after tumor exposure that translates into reduced monocyte activation and cytokine release. Importantly, this feature is intrinsic to CAR T cell products generated from T_N/SCM_ and independent of the costimulatory domain included in the CAR construct, offering a general way for developing CAR T cell therapies with ameliorated therapeutic indexes.

### CAR T_N/SCM_ fine-tune monocyte activation and proinflammatory cytokine production.

**Figure 6:**
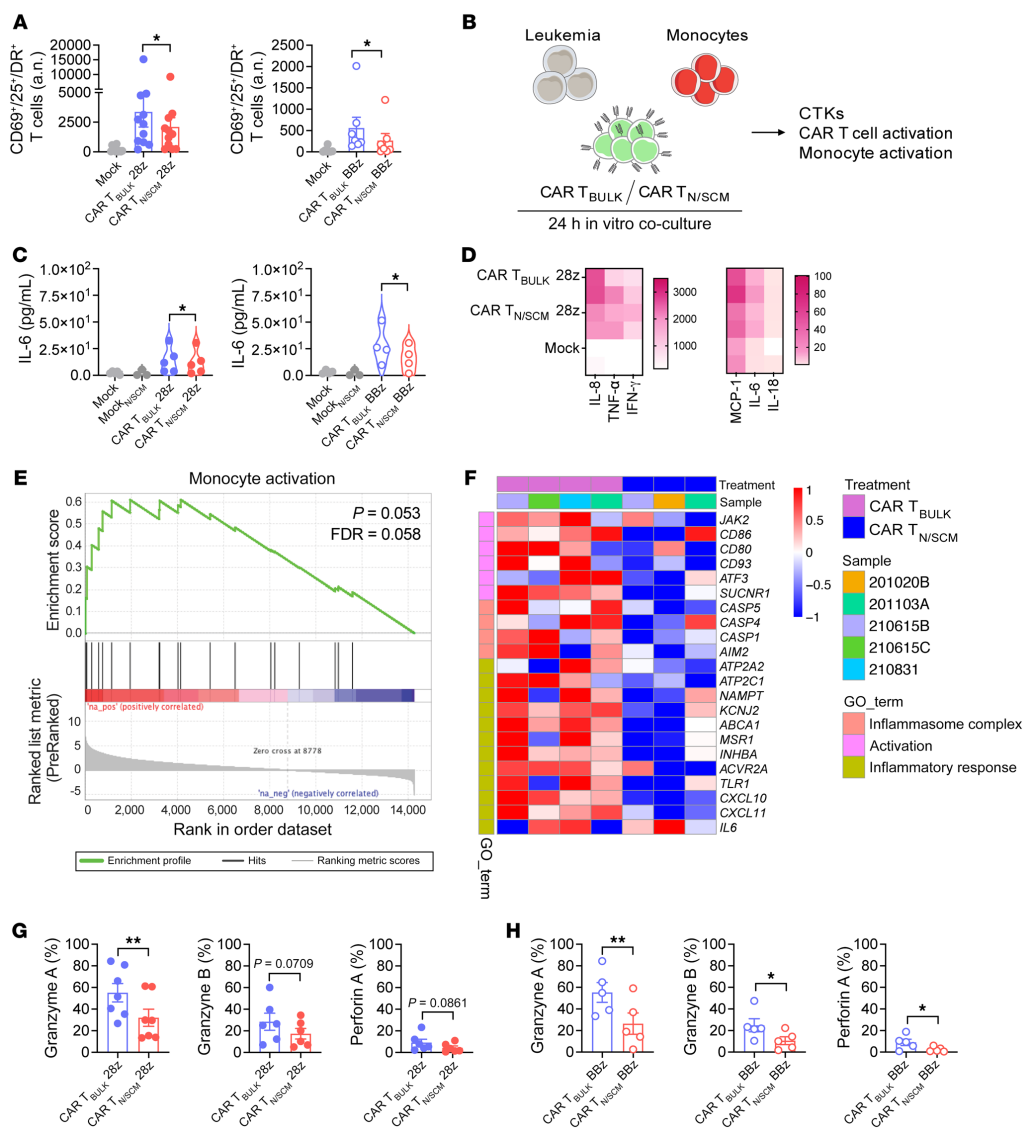
CAR T_N/SCM_ better calibrate monocyte activation and cytokine production. (**A**) Absolute number (a.n.) of CAR T cells coexpressing activation markers (CD25, CD69, HLA-DR) 24 hours after coculture with NALM-6 cells (CAR T_BULK_/CAR T_N/SCM_ 28z *n =* 11, left; CAR T_BULK_/CAR T_N/SCM_ BBz *n =* 8, right). (**B**) Schematic representation of tripartite cocultures consisting of NALM-6 leukemia cells, CAR T cells, and autologous monocytes. Untransduced T_BULK_ (Mock) and T_N/SCM_ (Mock_N/SCM_) were used as controls. CTKs, cytokines. (**C**) IL-6 production (Mock *n =* 3; Mock _N/SCM_ *n =* 3; CAR T_BULK_/CAR T_N/SCM_ 28z *n =* 5, left; CAR T_BULK_/CAR T_N/SCM_ BBz *n =* 4, right) and (**D**) heatmap visualization of cytokine release 24 hours after plating. *P* = 0.0319 for the comparison between CAR T_BULK_ BBz and CAR T_N/SCM_ BBz in **D**. (**E** and **F**) RNA sequencing analysis of monocytes retrieved from tripartite cocultures including 4-1BB–costimulated CAR T cells and analyzed by RNA sequencing. (**E**) Pre-ranked GSEA depicting the expression profile of monocytes employing the activation gene set GSE5099 (CAR T_BULK_ *n =* 4, CAR T_N/SCM_ *n =* 3). (**F**) Heatmap illustrating expression values (log_2_-transformed RPKM) of selected genes retrieved from different pathways in monocytes as inflammatory response, activation, and inflammasome complex. Percentage of (**G**) CD28- and (**H**) 41BB-costimulated CAR T cells expressing granzyme A, granzyme B, and perforin 24 hours after coculture with NALM-6 cells (CAR T_BULK_/CAR T_N/SCM_ 28z *n =* 6/7, CAR T_BULK_/CAR T_N/SCM_ BBz *n =* 5). Data are represented as mean ± SEM together with overlapping scattered values and box and violin plots. *P < 0.05; **P < 0.01 by paired *t* test (**A**, **C**, **D**, **G**, and **H**).

To better decipher the mechanisms underlying the peculiar behavior of CAR T_N/SCM_, we first evaluated CAR T cell activation responses and kinetics in vitro after stimulation with NALM-6 leukemia cells. Interestingly, CAR T_N/SCM_ cells including either the CD28 or the 4-1BB costimulatory domain activated less intensely than CAR T_BULK_, both in terms of CD25, CD69, and HLA-DR upregulation, even though the kinetics were superimposable between the 2 populations (Supplemental Figure 6, A–C and E–G). Moreover, when looking at CD25^+^CD69^+^HLA-DR^+^ triple-positive marker expression, we found that the amount of activated CAR T_N/SCM_ was significantly lower both at 24 (Figure 6A) and 48 hours after stimulation (Supplemental Figure 6, D and H).

To assess whether reduced activation could play a role in downscaling monocyte activation and cytokine production, we set up a tripartite coculture consisting of NALM-6 leukemia cells, CAR T cells, and autologous monocytes (Figure 6B). Similar to what we observed in vivo, production of IL-6 (Figure 6C) and other myeloid-derived cytokines (Figure 6D) was significantly reduced with CAR T_N/SCM_ compared with CAR T_BULK_, both in the case of CD28 and 4-1BB costimulation. Even in the presence of myeloid cells, CAR T_N/SCM_ were characterized by milder activation compared with CAR T_BULK_ (Supplemental Figure 7). To gain mechanistic insights into the differential activation of the myeloid compartment, we retrieved monocytes from tripartite cocultures with 4-1BB–costimulated CAR T_BULK_ or CAR T_N/SCM_ and analyzed their transcriptional profile by RNA sequencing. According to the in vivo data, monocytes from cocultures with CAR T_N/SCM_ were characterized by a lower activation state (Figure 6E) and a milder inflammatory signature (Figure 6F). Interestingly, among the genes upregulated by monocytes in the presence of CAR T_BULK_, we found those implicated in the activation of the inflammasome, which regulates the proteolytic maturation of IL-1β and IL-18. Inflammasome activation in myeloid cells has recently been implicated in the development of CAR T cell–associated CRS, as a consequence of tumor cell pyroptosis induced by CAR T cell–released granzyme B (43) and granzyme A (44). Remarkably, we observed that CAR T_N/SCM_ stimulated with NALM-6 cells produce lower levels of granzyme A, granzyme B, and perforin than CAR T_BULK_, both in the case of CD28 (Figure 6G) and 4-1BB costimulation (Figure 6H). These data suggest that this pathway may be implicated in the different abilities of CAR T_N/SCM_ and CAR T_BULK_ to activate myeloid cells and cause sCRS.

Results obtained with primary monocytes confirmed those achieved with the human leukemia monocytic cell line THP-1 (Supplemental Figure 8) and integrated the observations made in vivo. Collectively, these findings show that CAR T_N/SCM_ regulates monocyte responses more safely than CAR T_BULK_. Moreover, they reveal a close relationship between CAR T cell and myeloid cell activation levels and suggest that by modulating CAR T cell activation it is possible to modify the triggering of myeloid cells to release cytokines and cause systemic toxicity.

### CAR T_N/SCM_ with a milder effector behavior in vitro can be generated from patients with B-ALL.

**Figure 7:**
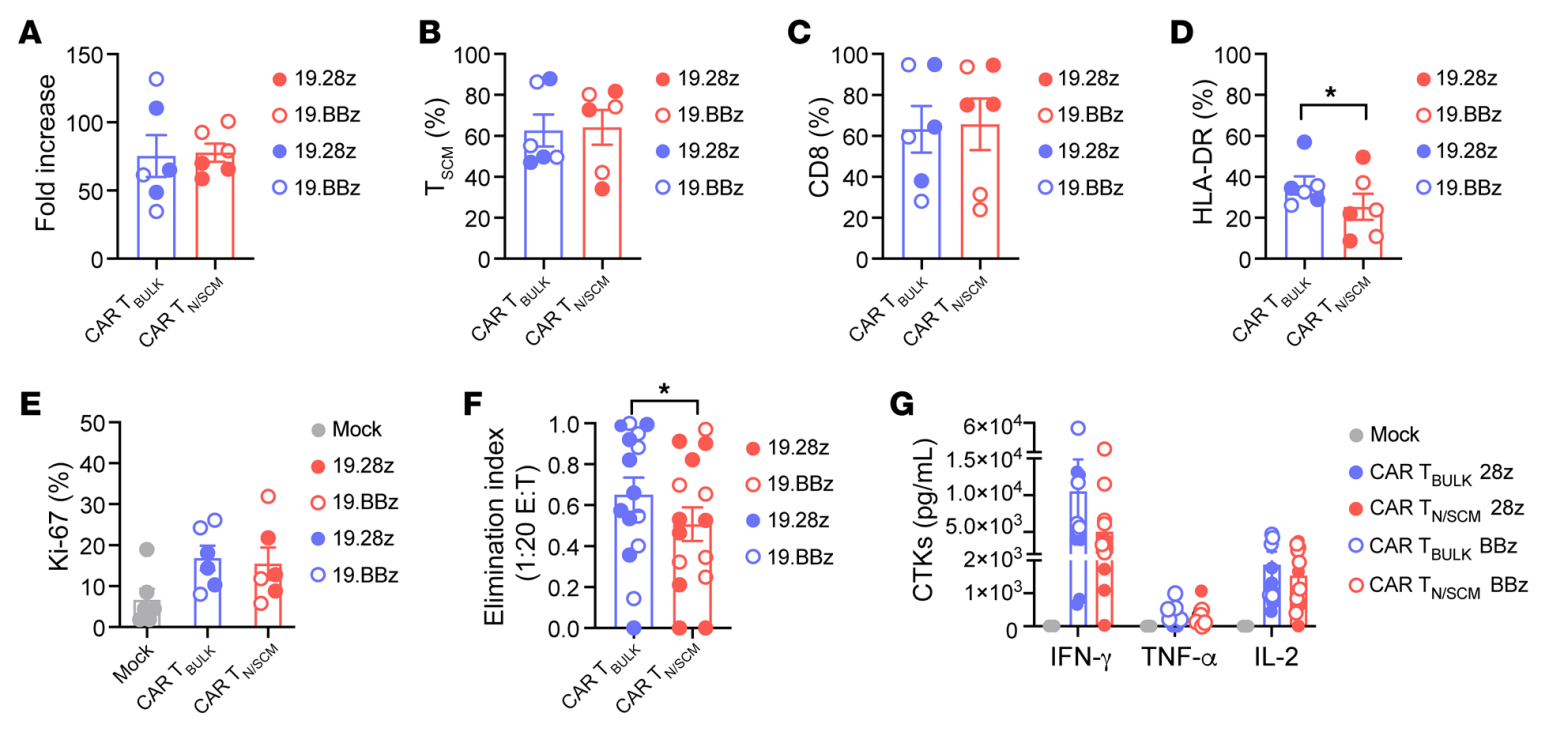
CAR T_N/SCM_ can be generated from patients with B-ALL. CD62L^+^CD45RA^+^ double-positive T cells from patients with B-ALL were isolated by FACS and bulk unselected T cells were employed as control. T_N/SCM_ and T_BULK_ were activated with TransAct, transduced with lentiviral vector encoding either a CD19.28z CAR or a CD19.BBz CAR, and expanded in culture with IL-7 and IL-15. (**A**) T cell fold expansion at the end of culture protocol (CAR T_BULK_/CAR T_N/SCM_ 28z *n =* 3, CAR T_BULK_/CAR T_N/SCM_ BBz *n =* 3). (**B**) T_SCM_ enrichment, (**C**) CD8^+^ frequency, and (**D**) HLA-DR expression at the end of manufacturing. (**E**) T cell proliferation after a 4-day coculture with NALM-6 cells, measured by intracellular staining of Ki-67. (**F**) Killing activity expressed as elimination index (see Methods) and measured by coculturing CAR T cells with NALM-6, BV173, and ALL-CM CD19^+^ tumor cells for 4 days at a 1:20 effector/target (E:T) ratio (CAR T_BULK_/CAR T_N/SCM_ 28z *n =* 9, CAR T_BULK_/CAR T_N/SCM_ BBz *n =* 6). (**G**) Cytokine (CTK) production after 24-hour coculture of CAR T cells with CD19^+^ cell lines at a 1:10 E:T ratio. Full circles refer to CAR constructs carrying the CD28 costimulatory domain, while open circles refer to CAR constructs carrying 4-1BB. Data are represented as mean ± SEM together with overlapping scattered values. *P < 0.05 by paired *t* test.

Since the above data suggest that CAR T_N/SCM_ display a higher therapeutic index compared with CAR T_BULK_, we aimed at demonstrating the feasibility of applying the preselection procedure, combined with a protocol capable of preserving T cell fitness, to T cells retrieved from 3 patients with B cell acute lymphoblastic leukemia (B-ALL). The mean percentage of T_N/SCM_ in these patients was 32.8% ± 7.6% (SEM), in line with clinical evidence (15, 45–48) and our previous findings (34) highlighting a lower frequency of these cell populations in B-ALL patients as compared with healthy donors. Nevertheless, CAR T_N/SCM_ were successfully generated in all cases, featuring high expansion rates at the end of the manufacturing protocol (Figure 7A). Phenotypic and functional characterization of patient-derived CAR T cells revealed a similar pattern to that of healthy donors. Both cell products were highly enriched in T_SCM_ (Figure 7B) and the CD4^+^/CD8^+^ ratio tended toward CD8^+^ (Figure 7C). However, CAR T_N/SCM_ featured lower activation levels at the end of manufacturing (Figure 7D) and, despite equal proliferation in short-term in vitro assays (Figure 7E), this cell product was slightly less cytotoxic against CD19^+^ cell lines than CAR T_BULK_ (Figure 7F) and released lower levels of inflammatory cytokines (Figure 7G).

Overall, these results provide proof of concept that applying optimized manufacturing protocols to preselected T_N/SCM_ cells allows generation of early memory CAR T cell products with a milder effector signature in vitro. This possibly translates into a favorable in vivo behavior both in terms of efficacy and toxicity profile.

## Discussion

CAR T cell fitness and antitumor activity can be enhanced through the enrichment of early memory subsets in the final cell product, by exploiting optimized manufacturing protocols (15, 20, 26, 34). However, whether preselecting specific T cell populations before manipulation would be really beneficial is still an open issue, due to the paucity of comprehensive in vivo data and lack of exhaustive toxicity profiling. Moreover, so far, the majority of studies have compared memory T cell subsets with each other and not with total T lymphocytes, which are the principal cell source employed in clinical trials. Even when T_BULK_ were considered as reference, stimulation with manufacturing protocols principally relying on OKT-3 and IL-2, which proved suboptimal in the capacity of generating long-lasting early memory T cells, were employed (20, 30, 49). In this work, we adapted the HSPC-humanized mouse model we recently developed (32) to investigate the efficacy and safety profiles of CAR T cells generated from preselected T_N/SCM_ or T_BULK_ employing a gold-standard procedure, based on stimulation with an anti-CD3/anti-CD28 nanomatrix and culture with IL-7/IL-15. Compared with the standard NSG mice, the HSPC-humanized model is characterized by the presence of innate immune cells and cytokines, offering thus a unique human network to uncover the full antitumor potential and safety profile of different CAR T cell populations.

We here show that, while being less potent in vitro, CAR T_N/SCM_ mediate more durable antitumor responses in HSPC-humanized mice compared with CAR T cell products generated from CAR T_BULK_. Improved activity was accompanied by higher expansion rates, which allowed unbalancing the effector/target ratio (E:T) in favor of T cells. Of note, despite fighting the tumor for several days, highly proliferating CAR T_N/SCM_ maintained a relevant pool of early memory T cells, displayed limited expression of IRs, and showed higher activation levels compared with CAR T_BULK_. We interpreted this result as improved fitness of CAR T_N/SCM_, which indeed proved uniquely able to counteract leukemia rechallenge in mice, envisaging an increased ability to protect patients from tumor relapse. In contrast, CAR T_BULK_ at the end of the first response were found to express multiple IRs at the expense of activation, suggesting that these cells have recognized the tumor and have become activated, but possess a limited propensity to guide full antitumor responses in the second challenge.

High CAR T cell expansion has been associated with increased incidence and severity of CRS and ICANS in patients (13, 17–19). Unexpectedly, however, CAR T_N/SCM_ showed a limited capability to induce severe toxicity, with negligible occurrence of grade 4 CRS and the majority of mice developing grade 1 or even no CRS (~66%). In contrast, CAR T_BULK_ induced grade 4 CRS in a significant proportion of mice (~30%) and only few had grade 1 CRS or remained CRS free (~20%). A clinical correlate to this finding is the observation that the employment of unselected CD8^+^ T cells compared with sorted T_CM_ CD8^+^ cells for CAR T cell manufacturing was associated with an increased risk of developing sCRS (17, 21). In keeping with this, it has been recently shown that heterogeneity of CAR T cell products further associates with variation not only in efficacy but also with regard to toxicity, especially in the case of CRS and ICANS development (24).

Importantly, we also observed that mice receiving CAR T_BULK_ and experiencing sCRS showed multifocal brain hemorrhages, which were absent in mice treated with CAR T_N/SCM_. Being similar to the events described in patients suffering from severe neurotoxicity in clinical trials, we interpreted these manifestations as signs of ICANS, resulting from endothelial damage (18, 19). These observations are quite interesting, although still preliminary, and fuel further investigation into the suitability of the HSPC-humanized model for studying ICANS development and pathogenesis, which is currently underway in our laboratory.

Interestingly, while CRS and neurotoxicity induction by CAR T_BULK_ was dependent on the tumor burden and T cell dose, CAR T_N/SCM_ proved to be intrinsically safer, independently of CAR costimulation, offering a unique option to limit patients’ risk of developing fatal toxicities while increasing efficacy.

It is known that endodomain costimulation dramatically influences CAR T cell fitness, with CD28 imprinting a prominent effector signature and 4-1BB inducing enhanced persistence and reduced differentiation (4, 50, 51). Our data suggest that CAR T_N/SCM_ are intrinsically less toxic, independently of the costimulation provided. Therefore, the choice of the most suitable costimulatory domain may presumably be undertaken depending on the context. For example, coupling the self-renewal potential of T_N/SCM_ with the typical effector capabilities of CD28 and its lower sensitivity to antigen density compared with 4-1BB (52) could provide the right balance to increase long-term persistence, without threatening efficient and rapid tumor debulking when dealing with solid malignancies or tumors expressing low antigen levels.

Toxic manifestations and antitumor activity are the result of complex pleiotropic and contact-dependent interactions taking place between activated CAR T cells and innate immune cells, with monocytes being primarily involved in the pathogenesis of both CRS and ICANS (32, 41). We thus hypothesized that CAR T_N/SCM_ inferior, yet progressive activation was capable of stimulating innate immune cells at sufficient levels for mediating supportive antitumor activity, without triggering detrimental side effects. Accordingly, we observed that CAR T_N/SCM_ are activated to a lesser extent than CAR T_BULK_ immediately after tumor encounter, resulting in milder monocyte activation and reduced cytokine production both in vivo and in tripartite cocultures including autologous monocytes. Particularly interesting was the observation that among the genes upregulated by monocytes in the presence of CAR T_BULK_ compared with CAR T_N/SCM_ we found those implicated in the activation of inflammasomes. These multimolecular complexes are known for their ability to control activation of caspase-1, which in turn regulates the proteolytic maturation of IL-1β and IL-18. Inflammasome activation in myeloid cells has recently been implicated in the development of CRS, as a consequence of tumor cell pyroptosis induced by CAR T cells (43). This form of proinflammatory cell death can be induced by the cleavage of gasdermin E and B by granzyme B and A, respectively, which are delivered into tumor cells via the action of perforin (43, 44). Intriguingly, we observed that CAR T_N/SCM_ express significantly less granzyme A, granzyme B, and perforin than CAR T_BULK_. These observations suggest that this pathway, which adds dying tumor cells as a new player in the interplay between CAR T cells and monocytes, could at least partially explain the lower toxic potential of CAR T_N/SCM_ as compared with CAR T_BULK_. However, further experiments will be needed to investigate this hypothesis in more detail.

Recent data suggest that diminishing signal strength in CAR T cells can result in lower toxicity and enhanced antitumor activity (53–55). Based on their indolent functionality, we hypothesized that CAR T_N/SCM_ were capable of differently processing the signal strength delivered by the CAR molecule per se, thus resulting in improved efficacy and safety profiles. Indeed, we found that a positive correlation exists between CAR T cell and monocyte activation, with CAR T_N/SCM_ featuring a reduced activation profile with both the CD28 and 4-1BB costimulatory domains. In this way, selectively manipulating sorted T_N/SCM_ should result in a final CAR T cell product endowed with superior expansion potential but lower activation aptitude, capable of better calibrating the dynamic cellular and molecular mediators responsible for sCRS development.

It has been reported that the frequency of T_N/SCM_ in heavily pretreated cancer patients can be extremely variable (15, 45–48). However, the preselection step could be highly beneficial to remove dysfunctional T cells, increasing CAR T cell quality and lowering the dose required to achieve antitumor efficacy (31). In this work, we provide proof of principle on the feasibility of applying this procedure to T cells retrieved from patients with B-ALL. Despite initial low frequencies of T_N/SCM_, CAR T_N/SCM_ showed extremely high expansion rates and a milder effector signature in vitro, as already observed for healthy donors. Further supporting the feasibility of this approach in cancer patients, we report that another group has already developed a clinical-grade procedure for preselecting T_N_ cells (20). Of note, the superiority of CAR T_N/SCM_ could also be exploited in the allogeneic setting, thus overcoming patient-intrinsic T cell defects and ensuring widespread accessibility to therapy (56). In both scenarios, preselection of T_N/SCM_ could allow reducing patient-to-patient variability and better comparing the results among different clinical trials.

Taken together, our results indicate that preselection of T_N/SCM_ can lead to a better balance between CAR T cell efficacy and safety profiles, significantly improving the therapeutic index of current CAR T cell therapies.

## Methods

### Cell lines.

Leukemic cell lines NALM-6 and BV173 were purchased from the American Type Culture Collection (ATCC) and cultured in RPMI 1640 (BioWhittaker) supplemented with 10% FBS (Lonza), 100 IU/mL penicillin/streptomycin, and glutamine. The ALL-CM cell line was provided by Fred Falkenburg (Leiden University Medical Center, Leiden, The Netherlands) and maintained in culture in X-VIVO (Lonza) with 3% human serum (Euroclone) and 100 IU/mL penicillin/streptomycin. For in vivo experiments, the NALM-6 cell line was transduced with a lentiviral vector encoding the secreted Lucia luciferase (Lucia^+^NGFR^+^ NALM-6), as previously reported (57).

### Transduction and culture conditions.

Buffy coats from healthy donors were obtained after written informed consent and IRB approval. CD45RA^+^CD62L^+^ T_N/SCM_ were isolated by FACS. B-ALL samples were selected on the basis of the disease classification (type B) and all patients received chemotherapeutic treatment. Patient-derived CD4^+^CD8^+^ T_BULK_ and CD4^+^CD8^+^CD62L^+^CD45RA^+^ T_N/SCM_ were isolated by FACS from peripheral blood mononuclear cells. T_BULK_ and T_N/SCM_, derived from either healthy donors or patients, were stimulated through MACS-GMP T Cell TransAct (Miltenyi Biotec) and transduced with a bidirectional lentiviral vector encoding either CD19.CAR.28z or CD19.CAR.BBz and the LNGFR marker gene. Bidirectional lentiviral backbones were provided by Luigi Naldini (San Raffaele-Telethon Institute for Gene Therapy, Milan, Italy). Cells were maintained in culture in TexMacs medium (Miltenyi Biotec), supplemented with low-dose IL-7/IL-15 (Miltenyi Biotec) for 15 days. Healthy donor CAR^+^ cells were enriched by sorting through magnetic labeling of the LNGFR marker gene. Phenotypic and functional analyses of each CAR T cell product were performed at the end of manufacturing.

### Multiparametric flow cytometry.

HuSGM3 peripheral blood samples were obtained on day 14 after CAR T cell infusion and stained with monoclonal antibodies specific for human CD3 BV605 (BioLegend, clone SK7, catalog 344836), CD8 BV650 (BD, clone SK1, catalog 565289), CD4 BUV496 L3T4 (BD, clone SK3, catalog 564651), CD57 BB515 (BD, clone NK-1, catalog 565285), CD223 (LAG-3) APC-R700 (BD, clone T47-530, catalog 565774), CD45RA APC-H7 (BD, clone HI100, 560674), TIGIT BV421 (BD, clone 741182, catalog 747844), CD279 (PD-1) BV480 (BD, clone EH12.1, catalog 566112), CD27 BV750 (BD, clone L128, catalog 747310), CD25 (IL-2 receptor α chain) BUV563 (BD, clone 2A3, catalog 612918), CD62L (L-selectin) BUV805 (BD, clone DREG-56, catalog 742024), CD95 (Fas/APO-1) PE-Cy 7 (BioLegend, clone DX2, catalog 305622), CD28 PE-Cy 5 (BD, clone CD28.2, catalog 555730), CD45 APC (BD, clone HI30, catalog 555485), CD272 (BTLA) BB700 (BD, clone J168-540, catalog 746166), CD197 (CCR7) PE (BD, clone 150503, catalog 562381), CD271 (NGF receptor) BUV395 (BD, clone C40-1457, catalog 743362), CD98 BUV661 (BD, clone UM7F8, catalog 750700), and CD154 BUV737 (BD, clone TRAP1, catalog 748983). Samples were stained in brilliant staining buffer (BD). In addition, CAR T cell and mouse samples were stained with one or more of the following conjugated monoclonal antibodies: CD3 PB (BioLegend, cloneHIT3a, catalog 300330), CD45 BV510 (BioLegend, clone HI30, catalog 304036), CD271 PE-Cy7 (BioLegend, clone CD40-1457, catalog 562122), CD271 PE (BD, clone C40-1457, catalog 557196), CD4 FITC (BioLegend, clone SK3, catalog 344604), mouse CD45 PerCP (BioLegend, clone 30-f11, catalog 103130), CD14 APC (BioLegend, clone M5E2, catalog 301820), CD19 APC/Cy7 (BioLegend, clone HIB19, catalog 302218), HLA-DR APC/Cy7 (BioLegend, clone L243, catalog 307618), CD45RA FITC (BioLegend, clone HI100, catalog 304106), CD62L APC (BioLegend, clone DREG-56, catalog 304810), CD8 PerCP (BD, clone SK1, catalog 345774), CD107a FITC (BD, clone H4A3, catalog 555800), Ki-67 Pacific Blue (BioLegend, clone KI67, catalog 350512), granzyme A Alexa Fluor 488 (BioLegend, clone CB9, catalog 507212), granzyme B Pacific Blue (BioLegend, clone GB11, catalog 515407), perforin A PE-Cy7 (BioLegend, clone B-D48, catalog 353315), CD69 APC (BioLegend, clone FN50, catalog 310910), CD25 APC/Cy7 (BioLegend, clone BC96, catalog 302614), CD163 FITC (BioLegend, clone GHI/61, catalog 333618), CD54 PE (BioLegend, clone HA58, catalog 353106), CD80 PE-Cy7 (BioLegend, clone 2D10, catalog 305218), and CD86 APC (BioLegend, clone IT2.2, catalog 305411). Flow cytometry data were acquired using BD FACSymphony and BD FACSCanto II cell analyzers and visualized with FlowJo v10 software.

### In vitro functional assays.

CAR T_BULK_ or CAR T_N/SCM_ cells were cocultured with CD19^+^ leukemic cell lines (Lucia^+^NGFR^+^ NALM-6, ALL-CM, BV-173) at different E:T ratios. Untransduced T cells were used as control (Mock). After 24 hours, supernatants were collected and analyzed with the LEGENDplex bead-based cytokine immunoassay (BioLegend). After 4 days, residual cells in culture were analyzed by FACS using Flow-Count Fluorospheres (BeckmanCoulter). The elimination index was calculated as follows: 1 − (number of residual target cells in presence of target antigen-specific CAR T cells/number of residual target cells in presence of control CAR T cells). For degranulation assays, T cells were labeled with FITC–anti-CD107a immediately after coculture with different CD19^+^ cell lines at a 1:3 E:T ratio. After 24 hours, cells were collected and analyzed by FACS. For proliferation assays, T cells were cocultured with CD19^+^ targets at a 1:1 E:T ratio. After 4 days, cells were stained for intracellular Ki-67 and analyzed by FACS. Concerning assays for CAR T cell activation kinetics, T cells and NALM-6 cells were cocultured at a 1:10 E:T ratio and CD69/CD25 upregulation together with HLA-DR expression were evaluated at several time points. Finally, a tripartite coculture consisting of NALM-6 leukemia, T cells, and autologous monocytes or THP-1 monocyte-like cells was conducted for 24 hours at a 1:1 E:T ratio. At the end of the experiment, supernatants were collected and analyzed as previously mentioned for cytokine detection, while the expression of CD163, CD86, HLA-DR, and CD54 activation markers was evaluated on T cells and monocytes as well as on monocyte-like cells. For granzyme A/B and perforin A assay, T cells were cocultured with CD19^+^ NALM-6 cells at a 1:1 E:T ratio. After 24 hours, cells were stained for intracellular granzyme A/B and perforin A and analyzed by FACS.

### In vivo experiments.

Six- to 8-week-old NSGTgCMV-IL3 CSF2 KITLG1Eav/MloySzJ (SGM3) mice (Charles River Laboratories) were sublethally irradiated and infused i.v. with 1 × 10^5^ human cord blood CD34^+^ cells (Lonza). Upon reconstitution, HuSGM3 mice were infused i.v. with 0.5 × 10^6^ Lucia^+^NGFR^+^ NALM-6 cells and 5 or 7 days later, in the low and high tumor burden setting, respectively, treated i.v. with 1 × 10^6^ or 1 × 10^7^ CD19^+^ CAR T_BULK_, CD19^+^ CAR T_N/SCM_, or control Mock T cells. Mice were sacrificed when relative light units (RLU) exceeded the threshold of 1.5 × 10^6^ or when manifesting clinical signs of suffering. For evaluating CRS development, weight loss was monitored daily and the concentration of serum human cytokines (LEGENDplex) and mouse SAA (ELISA kit, Abcam) were assessed weekly, according to the manufacturers’ instructions. CRS incidence and grading were calculated by taking into account several sCRS-related parameters, i.e., weight loss, death, together with IL-6, MCP-1, and IP-10 myeloid-derived cytokines, assigning a CRS grade to each treated mouse. Finally, the overall CRS score was represented by the sum of each parameter-associated score that was considered according to the level of statistical significance found between sCRS-related deaths and recovering animals.

### BH-SNE analysis.

BH-SNE was applied on concatenate downsampled CD3^+^ events (74,000 events/sample) collected from the peripheral blood of HuSGM3 NALM-6–bearing mice treated with CAR T cells, 14 days after infusion. The flow cytometry–based analysis was performed by downscaling surface proteome as cell surface markers. More precisely, exhaustion, memory, and activation markers were employed to calculate BH-SNE biaxial variables considering T lymphocytes (CD3^+^ events) as input. BH-SNE algorithm analysis settings were perplexity = 30,000 and theta = 0.5. The Flow-SOM algorithm was then applied for the cytometry variables of interest and clustered data into 50 different groups. Clusters were first studied in their composition by means of raw percentages and, when attributed to one experimental group, the mean fluorescence for the variables of interest was calculated and normalized according to the mean fluorescence of the total experimental data set.

### Histopathological analysis.

Brains from HuSGM3 mice were collected at necropsy, fixed in buffered 4% formalin, embedded in paraffin, cut and stained in the Good Laboratory Practice (GLP) SR-TIGET Pathology laboratory following GLP principles. Hematoxylin- and eosin-stained 3-μm paraffin sections were blindly and independently examined for histopathological analysis by 2 pathologists. Photomicrographs were taken using the AxioCam HRc (Zeiss) with the AxioVision System SE64 (Zeiss).

### RNA sequencing.

Monocyte RNA extraction was performed using a PicoPure RNA Isolation Kit (Thermo Fisher Scientific). Quality control (QC) check of all RNA samples was done by TapeStation HS RNA. All the samples were processed with a SMART-Seq v4 Ultra Low Input RNA Kit to ensure that the final cDNA libraries contain the 5′ end of the mRNA and maintain a true representation of the original mRNA transcripts. NGS library preparation for Illumina sequencing was performed with a Nextera XT DNA Library preparation kit. Limited-cycle PCR was used to amplify the insert DNA and to add index adapter sequences on both ends of the DNA as well. All of the samples were barcoded, pooled, and sequenced on an Illumina Nova-Seq 6000 sequencing system in single-read mode, obtaining an average of 30 million single-end reads 100 nt in length per sample. The raw reads produced from sequencing were trimmed using Trimmomatic v0.32 (https://github.com/usadellab/Trimmomatic/releases) to remove adapters and to exclude low-quality reads from the analysis. The remaining reads were then aligned to the human genome GRCm38 using STAR v2.5.3a (https://github.com/alexdobin/STAR/releases?page=3). Reads were eventually assigned to the corresponding genomic features using featureCounts, according to the Gencode basic annotations (Gencode v31; https://www.gencodegenes.org/human/release_31.html). Quality of sequencing and alignment was assessed by means of FastQC (https://github.com/s-andrews/FastQC/releases), RseQC (https://github.com/MonashBioinformaticsPlatform/RSeQC), and MultiQC (https://github.com/ewels/MultiQC/releases) tools. Expressed genes were defined as those genes showing at least 1 count per million reads (CPM) on at least a selected number of samples, depending on the size of the compared groups. Genes with low expression that did not match these criteria were excluded from the corresponding data set. Gene expression read counts were exported and analyzed in the R environment (v4.0.3) to identify differentially expressed genes (DEGs), using the DESeq2 Bioconductor library (v1.30.1; ref. 58). For FDR, *P* values were adjusted using a threshold for false discovery rate (FDR) of less than 0.05 (59); significantly DEGs were identified as those showing FDR less than 0.05. For sequencing QC (SEQC), significantly DEGs were identified as those showing a nominal *P* value of less than 0.01 and |log_2_(fold change)| of less than 1 (60). Functional enrichment analysis was conducted using the enrichR R package (v3.0) (61), starting from the lists of DEGs as defined by FDR less than 0.05. Pre-ranked gene set enrichment analysis (GSEA) (62) was performed for each DEG comparison, on all the expressed genes. The gene sets included in the GSEA were obtained from Canonical Pathways, Hallmark, and Gene Ontology (GO) collections as they are reported in the MSigDB database (http://www.gsea-msigdb.org/gsea/msigdb/collections.jsp Accessed December 2, 2021). The records have been deposited in NCBI’s Gene Expression Omnibus (GEO GSE200661).

### Statistics.

Statistical analyses were performed with Prism software v9.1.3 (GraphPad). Data are shown as mean ± SEM with at least *n =* 3 replicates. Data sets were analyzed with 2-tailed paired or unpaired Student’s *t* test, 2-way ANOVA, or Gehan-Breslow-Wilcoxon and Mantel-Cox 2-sided log-rank tests, depending on the experimental design. A *P* value of less than 0.05 was considered significant.

### Study approval.

All patients signed informed consent forms approved by the Ospedale San Raffaele Ethics Committee (Milan, Italy), in accordance with the Declaration of Helsinki. All mouse experiments were approved by the Institutional Animal Care and Use Committee (IACUC) of San Raffaele University Hospital and Scientific Institute (Milan, Italy) and by the Italian Governmental Institute of Health (Rome, Italy).

## Author contributions

SA, CM, and C Bove designed and performed experiments, analyzed data, interpreted results, and wrote the manuscript. FM designed and performed experiments and analyzed data. BC, LF, and REK performed experiments and analyzed data. FS, MP, and RN performed histopathological analysis. EB performed RNA sequencing data analysis and interpreted results. BG contributed scientific support for the experimental designs and scientific discussion. MAM provided active assistance in reviewing the manuscript and figure processing. M Carrabba provided patient samples and offered scientific support. FC, C Bonini, and AB contributed to the scientific discussion and manuscript revision. M Casucci designed the study, analyzed and interpreted the data, wrote the manuscript, and acted as senior author of the study.

## Supplementary Material

Supplemental data

## Version 1 05/03/2022

In-Press Preview

## Version 2 06/15/2022

Electronic publication
