# Supplementary material for: CAR T cell manufacturing from naive/stem memory T lymphocytes enhances antitumor responses while curtailing cytokine release syndrome
Source: J Clin Invest. 2022 Jun 15;132(12):e150807. doi: 10.1172/JCI150807 (PMC9197529; doi:10.1172/JCI150807)
Supplement: Supplemental data [file jci-132-150807-s121.pdf]

Supplementary Material

Figure S1. CAR $T_{N/SCM}$ are less differentiated and display a reduced exhausted-like status *in vitro*.

Figure S2. CAR $T_{N/SCM}$ expand more in HuSGM3 mice without causing detrimental side effects.

Figure S3. CAR $T_{N/SCM}$ and CAR T_{BULK} are effective *in vivo* and equally represented in the meta-cluster analysis.

Figure S4. CAR $T_{N/SCM}$ are less prone to cause multifocal brain hemorrhages *in vivo*.

Figure S5. CAR $T_{N/SCM}$ BBz display lower activation *in vitro* and expand more *in vivo*.

Figure S6. CAR $T_{N/SCM}$ activate less upon leukemia encounter *in vitro*.

Figure S7. Milder activation status characterizes CAR $T_{N/SCM}$ in the presence of myeloid cells.

Figure S8. CAR $T_{N/SCM}$ better calibrate monocyte-like activation and cytokine production.

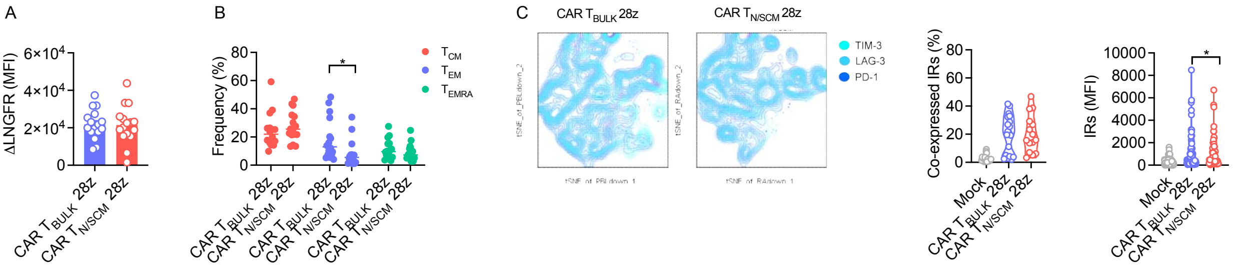

Figure S1. CAR $T_{N/SCM}$ are less differentiated and display a reduced exhausted-like status *in vitro*

A) ΔLNGFR marker expression reported as MFI at the end of culture protocol (n=16). **B**) Frequency of central memory, effector memory and terminally differentiated T-cell subsets at the end of culture (n=16). **C)** Left plots and panel: Tsne representation and percentage of T cells co-expressing TIM-3, LAG-3 and PD-1 inhibitory receptor (IRs) after co-culture with CD19+ targets (NALM-6 and ALL-CM cell lines; n=19 for CAR T_{BULK}, n=15 for CAR $T_{N/SCM}$). Right panel: IRs mean fluorescence intensity (MFI) values (n=11 donors). Data are represented as the result of mean ±SEM together with overlapping scattered values and box and violin plots. Results of two-way ANOVA (B) and paired t-test (A, C) are reported when statistically significant (*p<0.05).

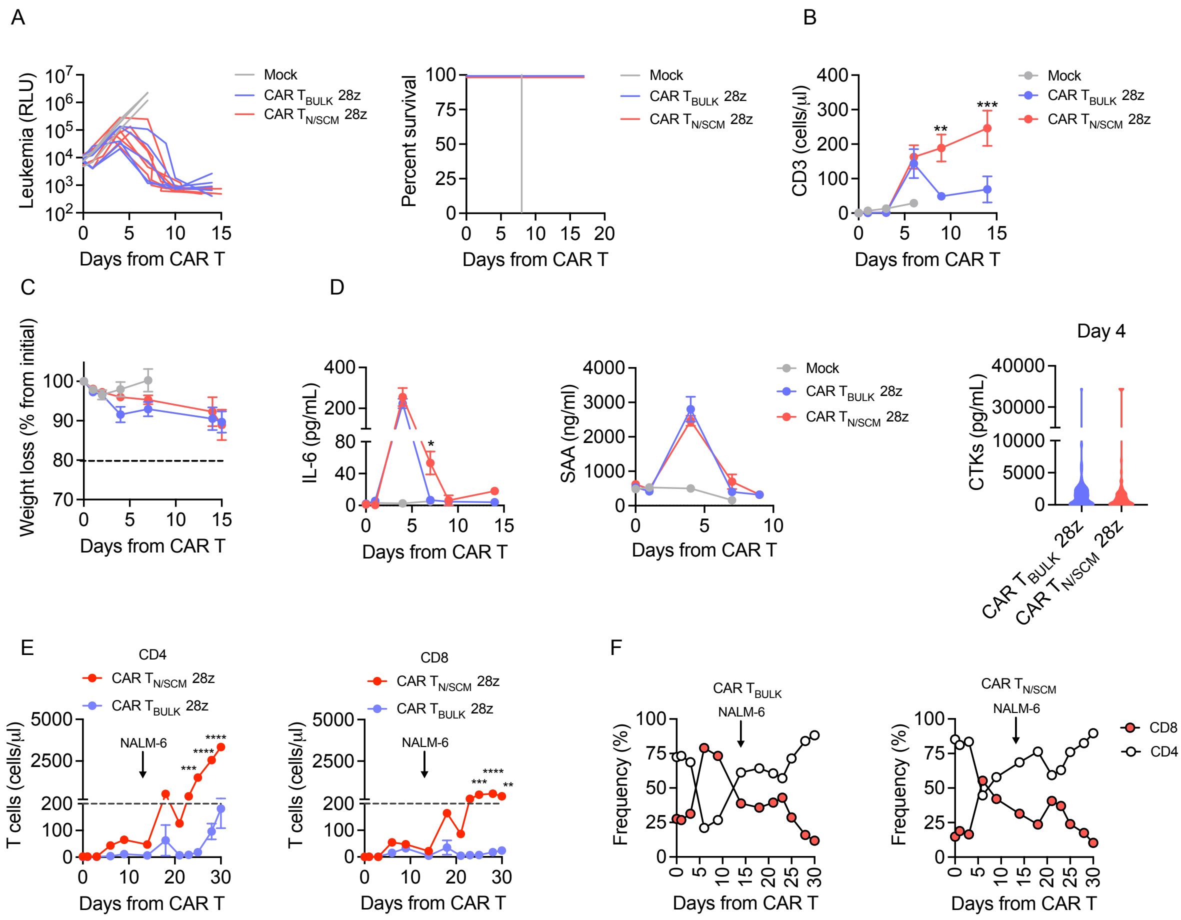

Figure S2. CAR $T_{N/SCM}$ expand more in HuSGM3 mice without causing detrimental side effects

HuSGM3 mice were infused with Lucia+/NGFR+/NALM-6 cells and treated with 3x10$^{\wedge 6}$ CAR $T_{N/SCM}$ (n=6), CAR T_{BULK} (n=5) or Mock control (n=3) and analyzed for antitumor activity and toxic manifestations. **A**) Left: Bioluminescence detection of tumor growth after treatment. Data are represented as single interspersed lines representing individual treated mice. Right: Kaplan–Meyer survival analysis of mice. **B**) T-cell expansion in the peripheral blood of mice. **C, D**) Evaluation of signs and symptoms typical of CRS development in HuSGM3 leukemia bearing mice, represented by weight loss (**C**) and serum levels of IL-6 (**D**, left), murine serum amyloid A (SAA, **D** middle) and other pro-inflammatory cytokines, namely IL-10, TNF-α, IL-1α, IFN-γ, MIP-1α, IP-10, MCP-1, IL-8, IL-2 and again IL-6 (**D**, right). **E**) CD4 (left) and CD8 (right) T-cell expansion within CAR T_{BULK} (n=17) and CAR $T_{N/SCM}$ (n=17) in the peripheral blood of NALM-6 bearing mice measured at

different time points after treatment. **F**) CD4 and CD8 frequency in CAR T_{BULK} (left) and CAR $T_{N/SCM}$ (right) measured in the peripheral blood of NALM-6 bearing mice at different time points after treatment. Data are represented as the result of mean ±SEM and box and violin plots. Results of unpaired t-test (D, right panel) and two-way ANOVA (A-F) are reported when statistically significant (*p<0.05; **p<0,01; ***p<0,001).

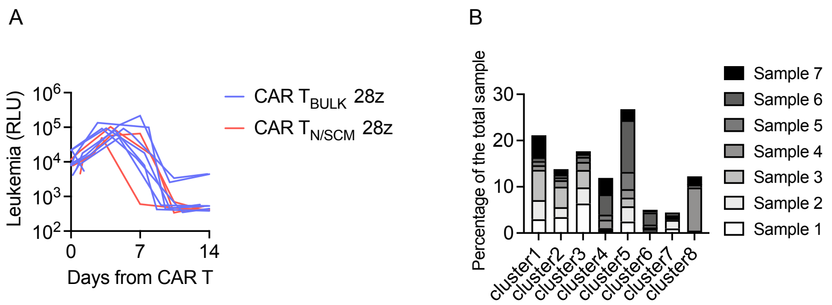

Figure S3. CAR $T_{N/SCM}$ and CAR T_{BULK} are effective *in vivo* and equally represented in the meta-cluster analysis

A) Bioluminescence detection of Lucia+/NGFR+/NALM-6 systemic growth in HuSGM3 mice after treatment with $1x10^{\wedge 6}$ CAR $T_{N/SCM}$, CAR T_{BULK} or Mock control. Data are represented as single interspersed lines representing individual treated mice (n=7 for CAR T_{BULK} and n=3 for CAR $T_{N/SCM}$).

B) Distribution of each sample in the relevant clusters after BH-SNE analysis.

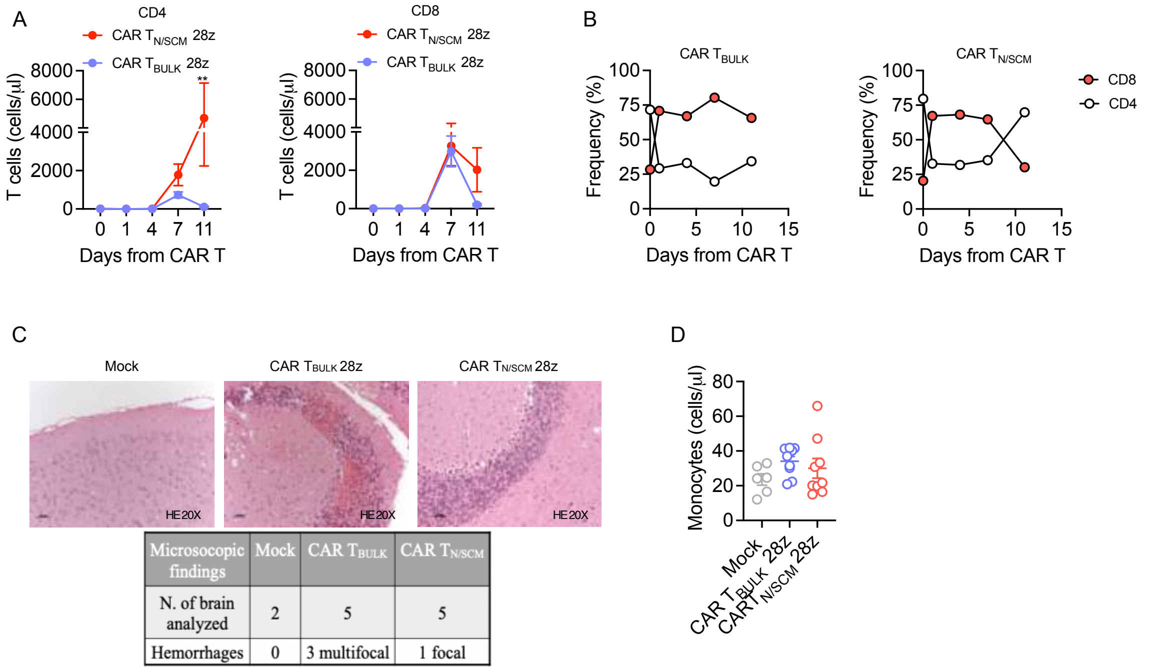

Microscopic findings	Mock	CAR T$_{BULK}$	CAR T$_{N/SCM}$
N. of brain analyzed	2	5	5
Hemorrhages	0	3 multifocal	1 focal

Figure S4. CAR T$_{N/SCM}$ are less prone to cause multifocal brain hemorrhages *in vivo*

A) CD4 (left) and CD8 (right) T-cell expansion within CAR T$_{BULK}$ (n=6) and CAR T$_{N/SCM}$ (n=6) in the peripheral blood of NALM-6 bearing mice measured at different time points after treatment. **B**) CD4 and CD8 frequency in CAR T$_{BULK}$ (left) and CAR T$_{N/SCM}$ (right) measured in the peripheral blood of NALM-6 bearing mice at different time points after treatment. **C**) Hematoxylin and eosin-stained section of brains belonging to representative Mock control, CAR T$_{BULK}$ and CAR T$_{N/SCM}$ treated mice (20x magnification; bar:50 micron) with relative incidence table of microscopic findings recorded in collected brains belonging to HuSGM3 leukemia-bearing mice treated with CD28-costimulated CAR T$_{N/SCM}$, CAR T$_{BULK}$ and Mock control. **D**) Monocyte absolute number immediately before CAR T-cell infusion in HuSGM3 leukemia bearing mice (n=9 for CAR T$_{BULK}$, n=9 for CAR T$_{N/SCM}$, n=6 for Mock).

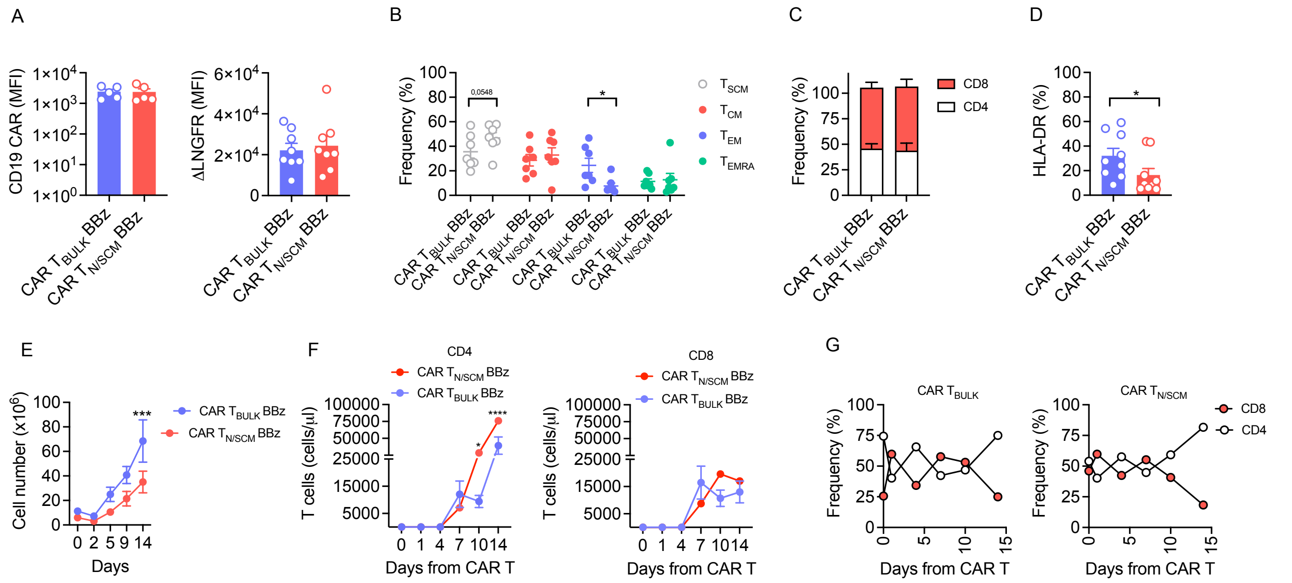

Figure S5. CAR $T_{N/SCM}$ BBz display lower activation *in vitro* and expand more *in vivo*

A) CD19.CAR scFv (n=5, left) and ΔLNGFR marker gene (n=8, right) expressed as MFI at the end of culture protocol. **B)** Memory phenotype at the end of T-cell manufacturing (n=7 donors). **C)** CD4/CD8 ratio (n=9 donors) and **D**) HLA-DR expression at the end of culture (n=9 donors). **E**) Fold expansion at different time points during culture (n=7). **F)** CD4 (left) and CD8 (right) T-cell expansion of CAR T_{BULK} (n=13) and CAR $T_{N/SCM}$ (n=12) in the peripheral blood of NALM-6 bearing mice measured at different time points after treatment. **G**) CD4 and CD8 frequency of CAR T_{BULK} (left) and CAR $T_{N/SCM}$ (right) measured in the peripheral blood of NALM-6 bearing mice at different time points after treatment. Data are represented as the result of mean ±SEM together with overlapping scattered values. Results of paired t-tests (C) and two-way ANOVA (A, B, D) are reported when statistically significant (*p<0,05; ***p<0,001).

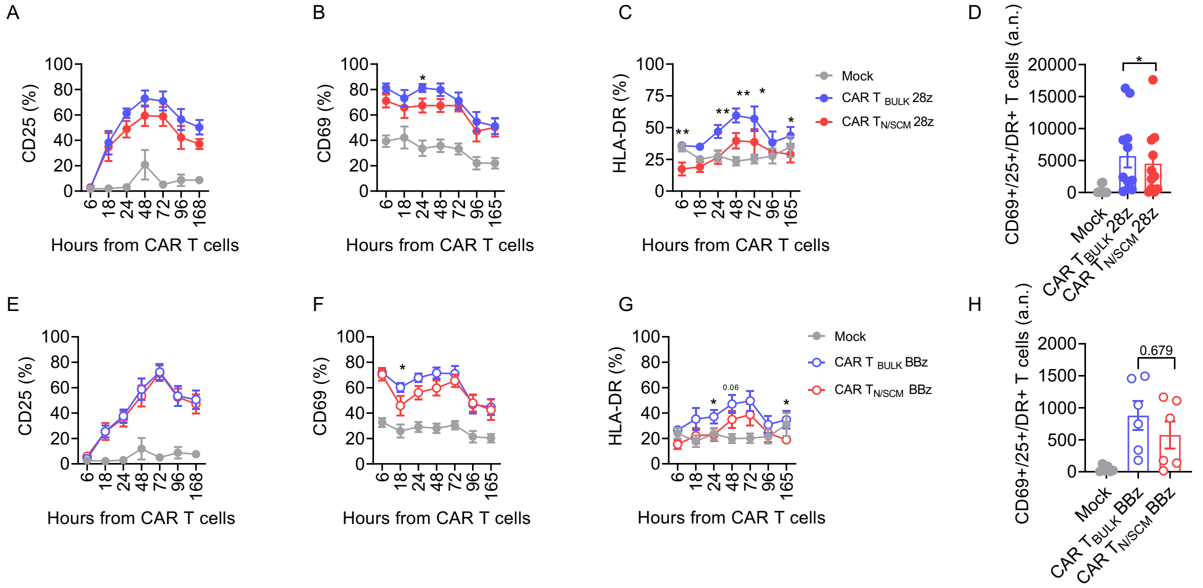

Figure S6. CAR $T_{N/SCM}$ activate less upon leukemia encounter *in vitro*

Activation kinetic of CAR T cells at different time points after stimulation with NALM-6 cells measured as upregulation of **A, E**) CD25 activation marker, **B, F**) CD69 activation marker and **C, G**) HLA-DR activation marker expression (CAR T_{BULK}/CAR $T_{N/SCM}$ 28z n=11; CAR T_{BULK}/CAR $T_{N/SCM}$ BBz n=11). **D, H**) Number of T cells co-expressing CD25/CD69/HLA-DR activation markers 48 hours after co-culture with tumor cells (n=11). Data are represented as the result of mean ±SEM together with overlapping scattered values and results of paired t-test is reported when statistically significant (*p<0,05).

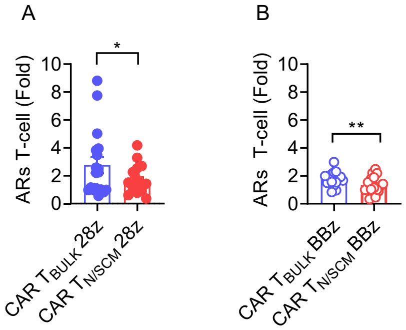

Figure S7. Milder activation status characterizes CAR $T_{N/SCM}$ in the presence of myeloid cells

Activation receptors (ARs) upregulation on **A**) CD28 and **B**) 4-1BB co-stimulated CAR T cells (CD54/CD86/HLA-DR/CD25) expressed as fold 24 hours after plating a tripartite coculture comprising NALM-6, CAR T cells and autologous monocyte (CAR T_{BULK}/CAR $T_{N/SCM}$ 28z n=5; CAR T_{BULK}/CAR $T_{N/SCM}$ BBz n=4).

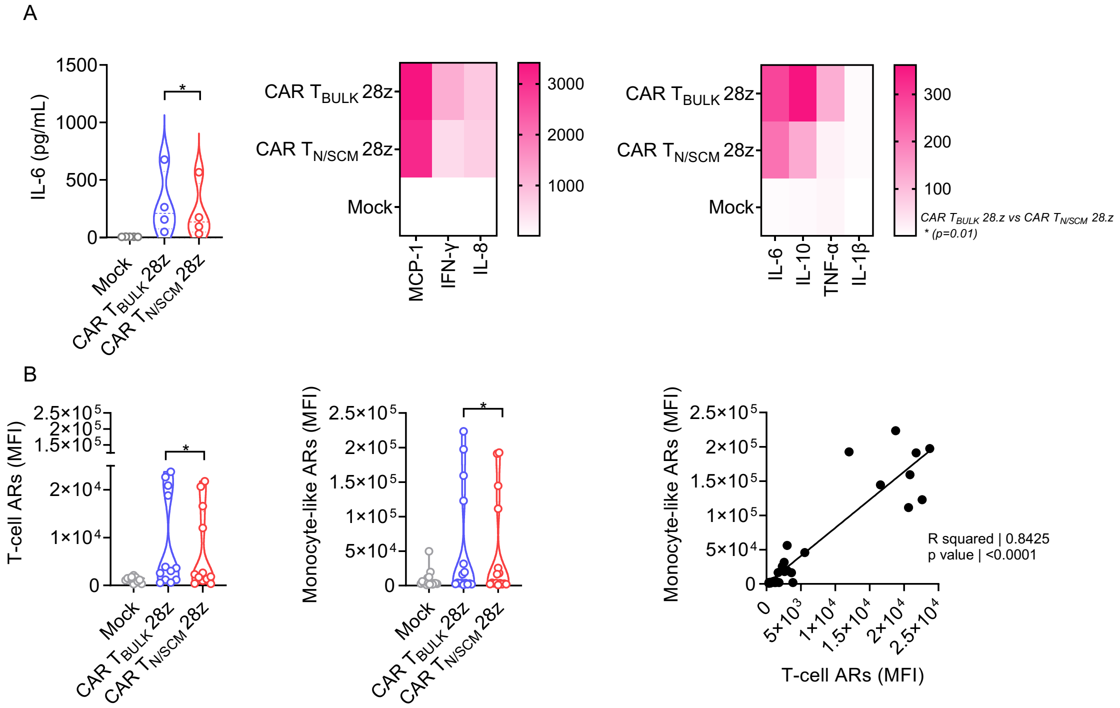

Figure S8. CAR $T_{N/SCM}$ better calibrate monocyte-like activation and cytokine production

CAR T_{BULK} and CAR $T_{N/SCM}$ were cultured with NALM-6 and THP-1 cells. **A**) IL-6 production (left panel) and heat-map visualization of cytokine release (right panel) 24 hours after plating (n=4). **B**) Activation receptors (ARs) upregulation on T cells (CD54/CD86/HLA-DR, left) and THP-1 cells (CD54/CD86/CD163/HLA-DR, middle) expressed as MFI 24 hours after plating, together with correlation analysis between T-cell and THP-1 activation states (considering CD54/CD86/HLA-DR, right; n=4).
